# Characterization of ozone in the lower troposphere during the 2016 G20 conference in Hangzhou

**DOI:** 10.1038/s41598-017-17646-x

**Published:** 2017-12-12

**Authors:** Wenjing Su, Cheng Liu, Qihou Hu, Guangqiang Fan, Zhouqing Xie, Xin Huang, Tianshu Zhang, Zhenyi Chen, Yunsheng Dong, Xiangguang Ji, Haoran Liu, Zhuang Wang, Jianguo Liu

**Affiliations:** 1 School of Earth and Space Sciences, University of Science and Technology of China, Hefei, 230026 China; 2 Key Lab of Environmental Optics & Technology, Anhui Institute of Optics and Fine Mechanics, Chinese Academy of Sciences, Hefei, 230031 China; 3 Center for Excellence in Regional Atmospheric Environment, Institute of Urban Environment, Chinese Academy of Sciences, Xiamen, 361021 China; 4 School of Atmospheric Sciences, Nanjing University, Nanjing, 210023 China

## Abstract

Recently, atmospheric ozone pollution has demonstrated an aggravating tendency in China. To date, most research about atmospheric ozone has been confined near the surface, and an understanding of the vertical ozone structure is limited. During the 2016 G20 conference, strict emission control measures were implemented in Hangzhou, a megacity in the Yangtze River Delta, and its surrounding regions. Here, we monitored the vertical profiles of ozone concentration and aerosol extinction coefficients in the lower troposphere using an ozone lidar, in addition to the vertical column densities (VCDs) of ozone and its precursors in the troposphere through satellite-based remote sensing. The ozone concentrations reached a peak near the top of the boundary layer. During the control period, the aerosol extinction coefficients in the lower lidar layer decreased significantly; however, the ozone concentration fluctuated frequently with two pollution episodes and one clean episode. The sensitivity of ozone production was mostly within VOC-limited or transition regimes, but entered a NOx-limited regime due to a substantial decline of NOx during the clean episode. Temporary measures took no immediate effect on ozone pollution in the boundary layer; instead, meteorological conditions like air mass sources and solar radiation intensities dominated the variations in the ozone concentration.

## Introduction

Recently, air pollution has become an increasingly serious environmental problem in China^1,2^. To protect the public health, many air quality control strategies have been being implemented by the Chinese government. As a result, ambient primary pollutants, such as sulfur dioxide (SO_2_)^3^ and nitrogen oxide (NO_x_ = NO + NO_2_)^4^, have begun to decrease. However, secondary pollutants such as secondary aerosols, which are dominant components of fine particulate matter (PM_2.5_), and ozone (O_3_) are still concentrated at high levels. Severe haze pollution events caused by PM_2.5_ still frequently occur in China, especially in the winter^5–7^. Moreover, tropospheric and ground-level O_3_ concentrations have displayed a rapidly increasing trend in China since the turn of the century^8,9^. Compared to PM_2.5_, the responses of ambient O_3_ to pollution emissions are more complex. In brief, O_3_ in the lower troposphere is mainly produced through the photochemical reactions between NO_x_ and volatile organic compounds (VOCs), but the interrelations among O_3_, NO_x_ and VOCs are complex and nonlinear^10–12^. However, field monitoring schemes for O_3_ and its precursors are still limited relative to those for PM_2.5_^9^. In particular, the vertical distributions of O_3_ in the troposphere and in the boundary layer are not uniform^13–17^, and boundary-layer O_3_ usually accumulates in the upper layer^18,19^. Observations of the vertical O_3_ structure are therefore essential for a comprehensive understanding of O_3_ pollution.

In recent years, a series of temporary and strict pollution control measures, such as the curbing or halting of production from power plants and factories, the limitation of vehicles and the prohibition of all construction activities, were implemented in China during some significant events, including the Asia-Pacific Economic Cooperation (APEC) summit in November 2014 and the Grand Military Parade in September 2015 in Beijing. These significant events provided natural laboratories for investigations of the influences of anthropogenic emissions on the air quality^20–22^. In September 2016, the conference for the Group of Twenty Finance Ministers and Central Bank Governors (G20) was held in the city of Hangzhou, the capital of Zhejiang Province. Hangzhou is located in the center of the Yangtze River Delta, one of the most developed areas in China. Similar to other significant events held in Beijing, strict pollution control measures were implemented in Hangzhou and its surrounding regions from Aug. 25 to Sep. 6. Here, we retrieve the vertical column densities (VCDs) for tropospheric O_3_, nitrogen dioxide (NO_2_) and formaldehyde (HCHO) from Aug. 14 to Sep. 18 based on Ozone Monitoring Instrument (OMI) satellite products. In particular, from Aug. 25 to Sep. 9, a ground-based ozone lidar was employed to monitor the vertical profiles of the O_3_ concentration and the aerosol extinction coefficients in the lower troposphere over urban Hangzhou (30.28° N, 120.13° E). To evaluate the effects of the control measures, we define the period from Aug. 26 to Sep. 6 as “G20”, while the periods before G20 during Aug. 14–25 and after G20 during Sep. 7–18 are defined as “pre-G20” and “post-G20”, respectively. Combing lidar and satellite data, the effects of pollution control measures on the air quality and the influencing factors on the lower tropospheric O_3_ concentration are investigated.

## Results and Discussion

### General signature of O_3_ and aerosol extinction

**Figure 1:**
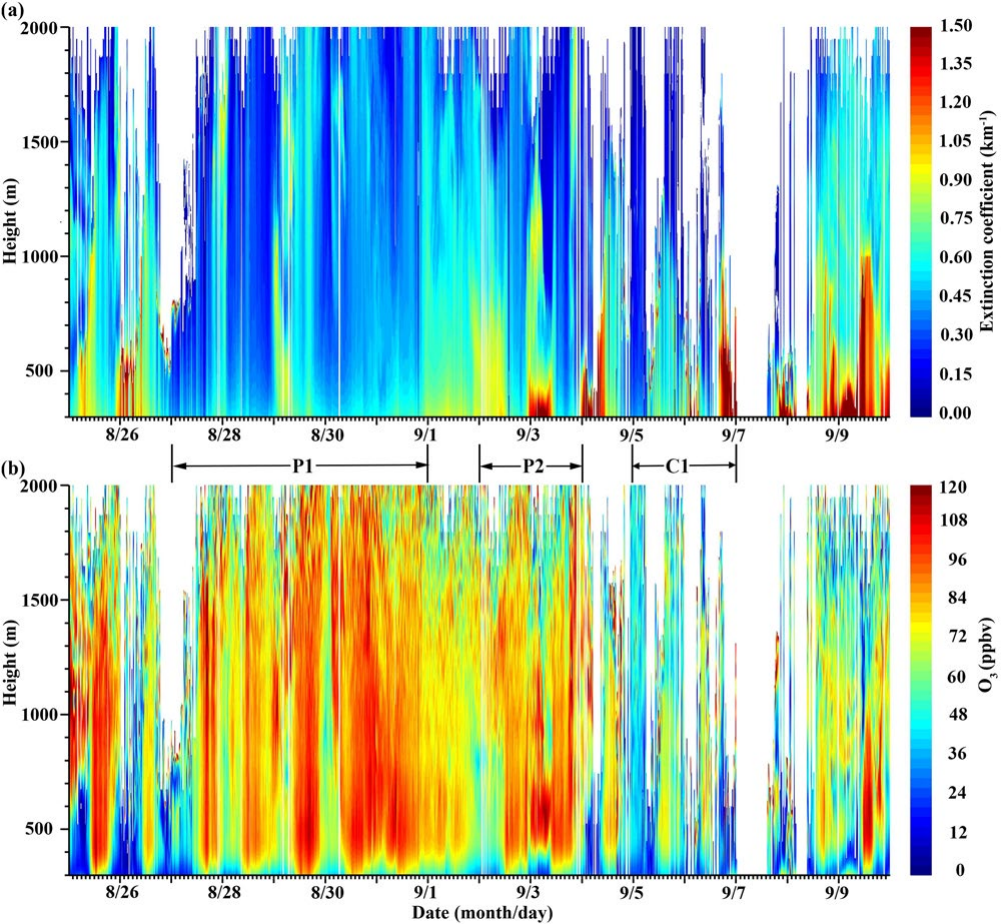
Time series of the vertical profiles for the (**a**) aerosol extinction coefficient and (**b**) O_3_ concentration measured by the ozone lidar in Hangzhou. A label on the x-axis of “8/26” represents a time of 00:00:00 on Aug. 26 (local time, UTC + 8).

Because the field of view between the laser and receiver does not completely overlap, vertical fade zones always exist in lidar observations^23–25^. For the ozone lidar employed in Hangzhou, the vertical fade zone is ~300 m. Owing to the decay of laser energy with increasing altitude, ~20% of the measured data above 2000 m had relative errors exceeding the lidar threshold value of 20% (Figure S1 in the Supplementary Information). Therefore, the lidar data for Hangzhou were adopted only between 300 m and 2000 m above ground level (AGL), and presented in Fig. 1. For the convenience of the following discussion, the vertical range of lidar observations is divided into three sections: the lower lidar layer (300–500 m) representing the lower to middle boundary layer, the middle lidar layer (500–1000 m) representing the upper boundary layer, and the upper lidar layer (1000–2000 m) representing the bottom of the free troposphere.

As displayed in Figure S2, after being integrated into the 1-hour resolution data, the O_3_ concentrations in the lower lidar layer were significantly positively correlated with the surface O_3_ acquired from two atmospheric environment automatic monitoring stations in Hangzhou: Xixi (30.27° N, 120.06° E), which is located 6.7 km from the lidar site (R = 0.69, P < 0.01), and Xiasha (30.31° N, 120.35° E), which is located 21 km from the lidar site (R = 0.71, P < 0.01). In addition, the aerosol extinction coefficients in the lower lidar layer showed a trend similar to the PM_2.5_ concentration at Xiasha (R = 0.68, P < 0.01). Although the relationship between the aerosol extinction coefficients and PM_2.5_ at Xixi was less robust (R = 0.46, P < 0.01), they generally also showed a coincident trend. In conclusion, although lidar cannot directly detect atmospheric components near the surface, measurements in the lower lidar layer can still reflect the trends of surface O_3_ and PM_2.5_. The O_3_ concentration during the research period was also simulated using the WRF-Chem model (Figure S3). At the lidar site, the simulated O_3_ showed a coincident trend with the lidar data in the lower and middle lidar layers, indicating that the model successfully reproduced the variations in O_3_ in the boundary layer (Figure S4). However, in the upper lidar layer, the observation and modelling results demonstrated a large deviation.

**Figure 2:**
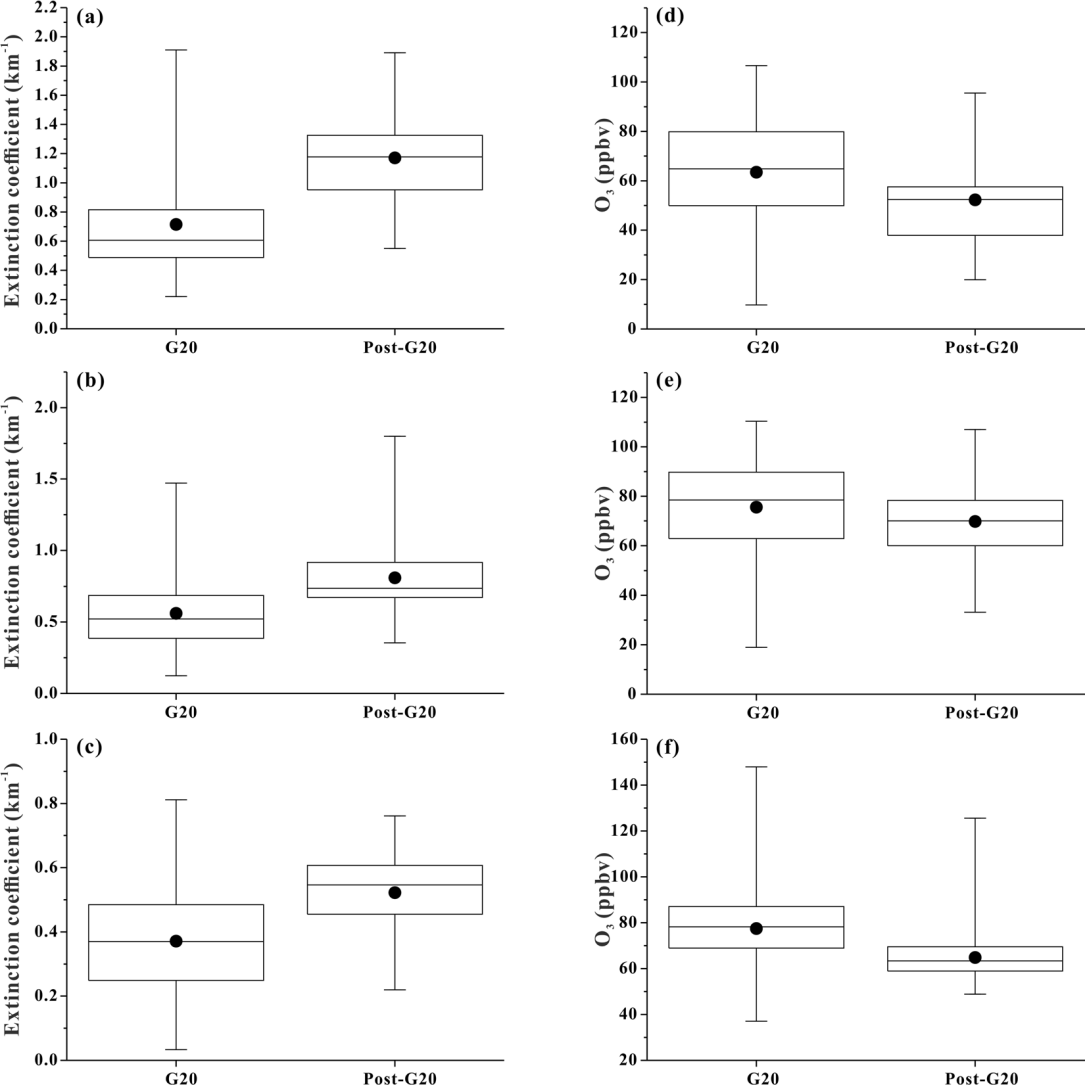
Box-and-whisker plots of the aerosol extinction coefficients during the G20 (Aug. 26-Sep. 6) and post-G20 (Aug. 8–9) periods in (**a**) the lower lidar layer, (**b**) the middle lidar layer and (**c**) the upper lidar layer; box-and-whisker plots of the O_3_ concentrations during the G20 and post-G20 periods in (**d**) the lower lidar layer, (**e**) the middle lidar layer and (**f**) the upper lidar layer. The lower and upper boundaries of the boxes represent the 25th and the 75th percentiles, respectively; the whiskers below and above the boxes indicate the minimum and maximum, respectively. The line within the box marks the median; while the dot represents the mean.

To evaluate the effects of pollution control measures, we compared the O_3_ concentrations and aerosol extinction coefficients measured using the ozone lidar during different periods. Owing to the limited quantity of data acquired during only one day, the lidar data during the pre-G20 period are not used for a comparison herein, and the data from Sep. 7 are also excluded because lidar data were unavailable for 13 hours on this day due to thick cloud cover. The aerosol extinction coefficients in the lower and middle lidar layers during the G20 period were significantly lower (P < 0.05) than those during the post-G20 period (Fig. 2a,b). Similarly, the surface PM_2.5_ concentrations at Xixi and Xiasha also increased during the post-G20 period (Figure S5). Pollution control measures therefore played a role in mitigating particle pollution in the boundary layer. Nevertheless, the difference between the aerosol extinction coefficients in the upper lidar layer during the two periods was not evident, and the mean level during the G20 period was even higher than that during the post-G20 period. However, the mean and median O_3_ levels in the lower, middle and upper lidar layers as well as the surface concentrations at Xixi and Xiasha during the G20 period were all higher than those afterwards (Fig. 2d,e,f). Thus, temporary emission control measures did not impose an immediate effect on O_3_ pollution.

### Diurnal Variations and Vertical Distribution

**Figure 3:**
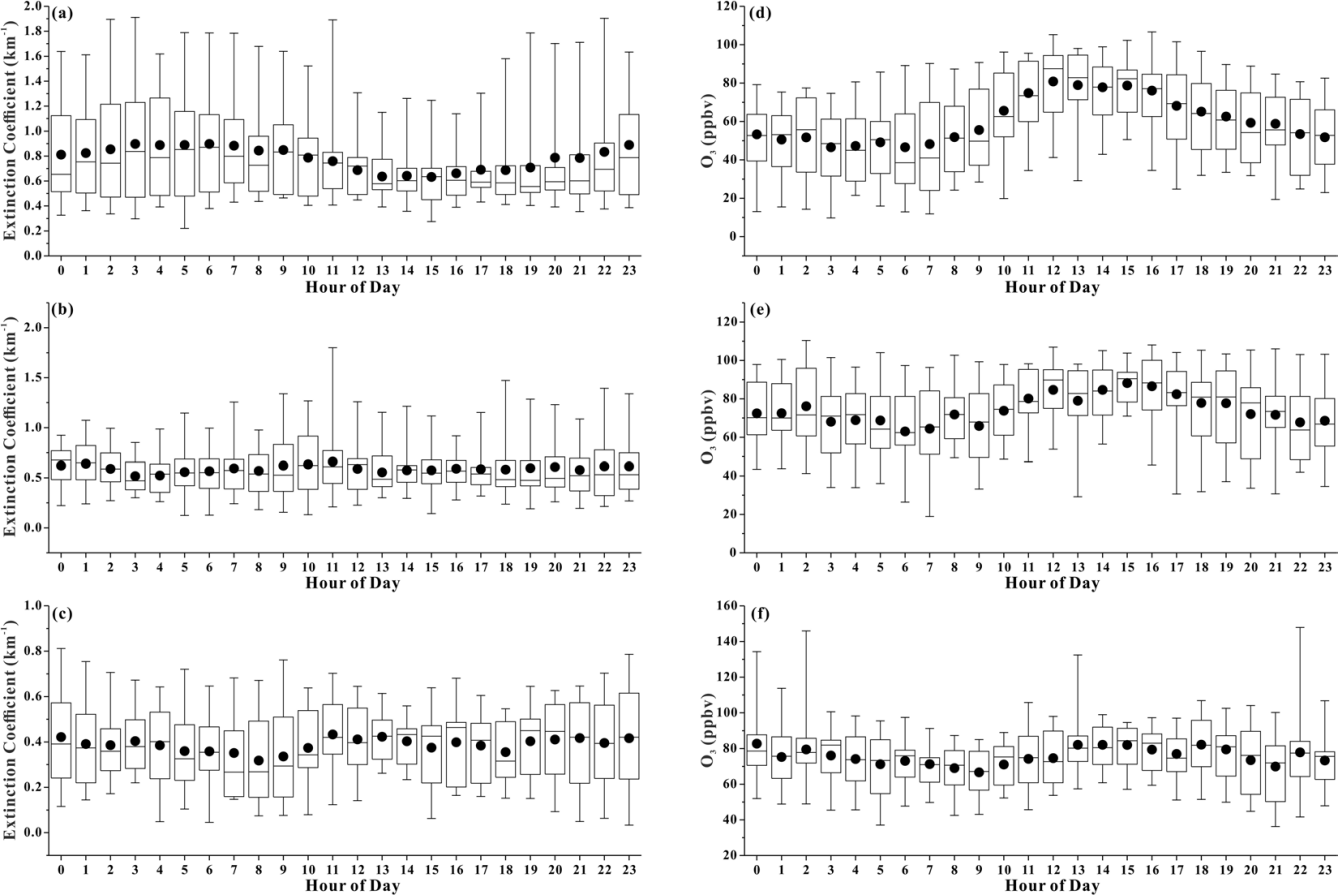
Diurnal variation box-and-whisker plots of the aerosol extinction coefficient in (**a**) the lower lidar layer, (**b**) the middle lidar layer and (**c**) the upper lidar layer; diurnal variation box-and-whisker plots of the O_3_ concentrations in (**d**) the lower lidar layer, (**e**) the middle lidar layer and (**f**) the upper lidar layer.

**Figure 4:**
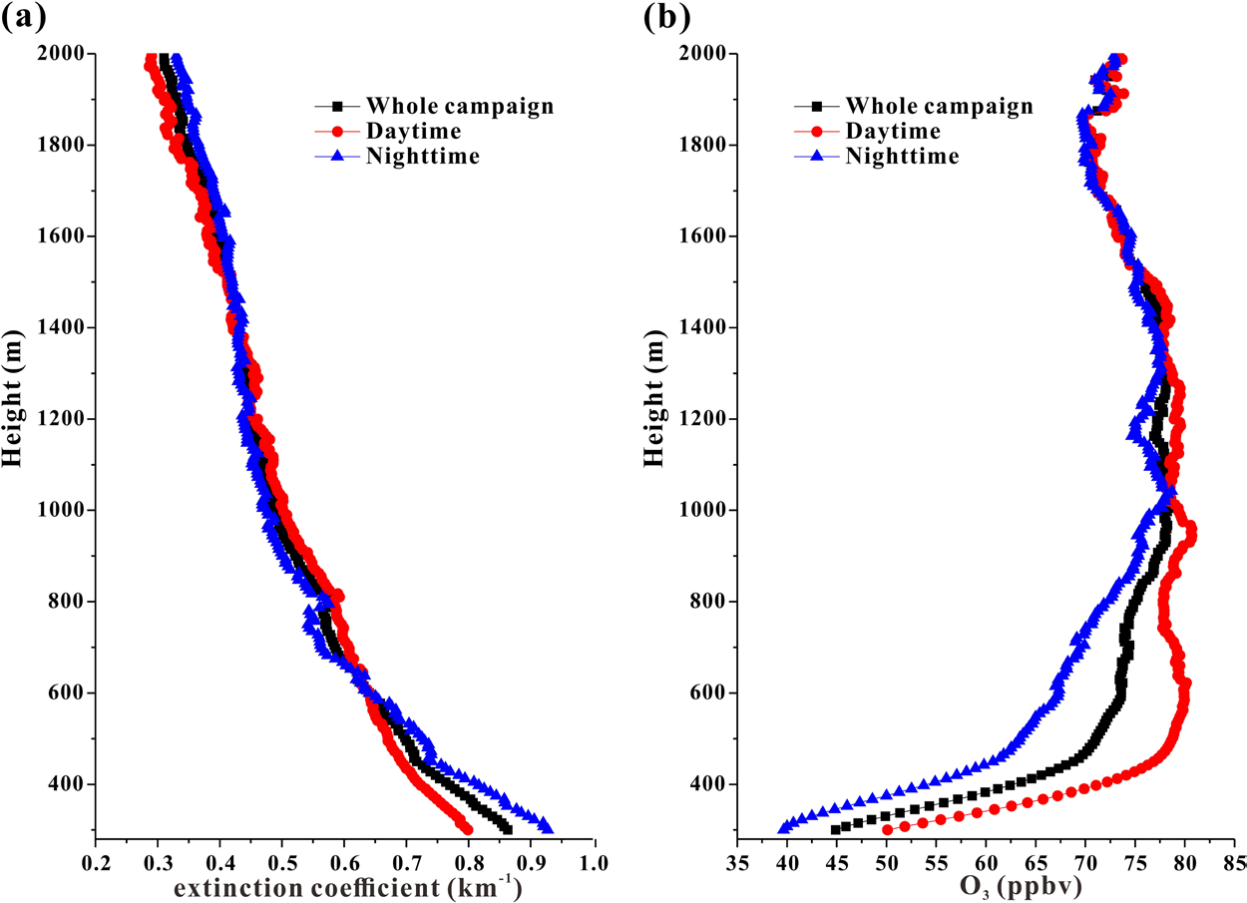
(**a**) Average vertical profiles of the aerosol extinction coefficient for the whole lidar campaign in addition to the daytime and the nighttime; (**b**) average vertical profiles of the O_3_ concentration for the whole lidar campaign in addition to the daytime and the nighttime.

Many air pollutants display diurnal variations owing to the diurnal cycles of their influencing factors, such as emissions, meteorological conditions and atmospheric chemical reactions. For instance, human activities and photochemical reactions are more intense during the daytime than during the nighttime, and would therefore induce higher levels during the daytime. However, higher boundary layer heights during the daytime would favor the diffusion of pollutants, and thus lower their concentrations. In this study, the aerosol extinction coefficients in the lower lidar layer during the nighttime (0.84 ± 0.41) were significantly higher than those during the daytime (0.73 ± 0.29) with peaks during 3:00–7:00 and at 23:00 and a trough during 13:00–15:00 (Fig. 3). Similarly, the surface PM_2.5_ at Xixi and Xiasha also reached a trough during approximately 13:00–15:00 (Figure S6a,b). This indicates a predominant role of diurnal cycles of the boundary layer height. However, in the middle and upper lidar layers, the peak values occurred during 9:00–11:00 and 10:00–14:00, respectively. Atmospheric turbulence is more intensive and the boundary layer rises during the late morning and noon, inducing the diffusion of more aerosols to higher altitudes from the surface. The average vertical profiles of the aerosol extinction coefficients for the whole lidar campaign in addition to the daytime and nighttime are presented in Fig. 4a. Overall, the aerosol extinction coefficients decreased with increasing altitude, confirming that the dominant sources of atmospheric particles are in the lower boundary layer.

The O_3_ in the lower lidar layer began to increase at approximately 8:00 in the morning with the onset of solar illumination and accumulated to a peak during 12:00–16:00 on most days (Fig. 3). Then, it began to decrease with fading solar radiation intensities. Similar diurnal patterns were also observed for the surface O_3_ at Xixi and Xiasha (Figure S6c,d). Generally, the diurnal cycle patterns of O_3_ in the lower- middle boundary layer was similar to that of the downward shortwave radiation (SWDOWN) flux at the ground surface simulated using the WRF-Chem model (Figures S2a and S6e). The peak of the O_3_ concentrations occurred approximately 1 hour behind that of the SWDOWN flux, which is similar to a discovery in Hong Kong, China^26^. During the lidar campaign, the average O_3_ concentration in the lower lidar layer in the daytime (7:00–18:00) was 69 ± 22 ppbv, which is significantly higher (P < 0.01) than the nighttime (19:00–6:00) concentration of 53 ± 19 ppbv. The O_3_ in the middle lidar layer also showed a clear diurnal variation with significantly higher concentrations during the daytime than during the nighttime and a peak during 12:00–16:00. However, there was no clear diurnal pattern in the upper lidar layer, and the mean levels during the nighttime and daytime were comparable. According to a previous study, the diurnal cycle of O_3_ is suppressed with an increase in the altitude, and no diurnal cycle is discernible above a certain height^27^.

The average vertical profiles of O_3_ during the daytime and nighttime were broadly agreeable (Fig. 4b). The O_3_ concentrations increased rapidly with an increase in the height below 500 m and continued to increase with slower rates to a peak at ~1000 m. Then, within ~1000–1800 m, the O_3_ concentrations generally exhibited a decreasing trend. A recent model investigation in Beijing during the summer also reproduced a peak at the top of the boundary layer (~1000 m)^18^. The O_3_ profile in the lower troposphere was mainly determined by the relative weights between the photochemical production and loss rates of O_3_. Typically, both of these rates decrease with increasing height, but the loss rate decreases more quickly at lower altitudes while the production rate decreases more quickly at higher altitudes^15^. The net O_3_ production, which represents a balance between the production and loss rates, reached a peak in the upper boundary layer. Furthermore, quick titration reactions of NO with O_3_ further lower O_3_ levels in the lower boundary layer^18,19,28^. As a result, the vertical profile of the O_3_ concentration first increased and then decreased with increasing altitude, similar to previous reports in Hong Kong^15^, Brazil^13^ and the U.S.^14^. Above 1800 m, the O_3_ concentrations increased again with increasing altitude, owing to air mass exchanges with the free troposphere, where the O_3_ concentrations are much higher than those in the boundary layer^17^.

### O_3_-NO_x_-VOCs sensitivities

The O_3_ concentrations in the lower troposphere is usually affected by *in-situ* photochemical reactions, regional transport^26^ and vertical injections from the free troposphere and stratosphere^29,30^. During Aug. 27–31 and Sep. 2–3, the O_3_ concentrations in the highest daily maximum 8-hour averages (DMA-8h) in the lower lidar layer were above the national level-II standard (GB-3095–2012) of 160 μg m^−3^ (~80 ppbv). Accordingly, on these days, the surface O_3_ at Xixi and Xiasha also presented high levels. After Sep. 4, the O_3_ concentrations started to decrease and stayed at low levels during Sep. 5–6. To explore the factors influencing the variations in the boundary-layer O_3_ concentrations, two pollution episodes, namely, P1 (Aug. 27–31) and P2 (Sep. 2–3), and a clean episode labeled C1 (Sep. 5–6) were identified during the lidar campaign.

**Figure 5:**
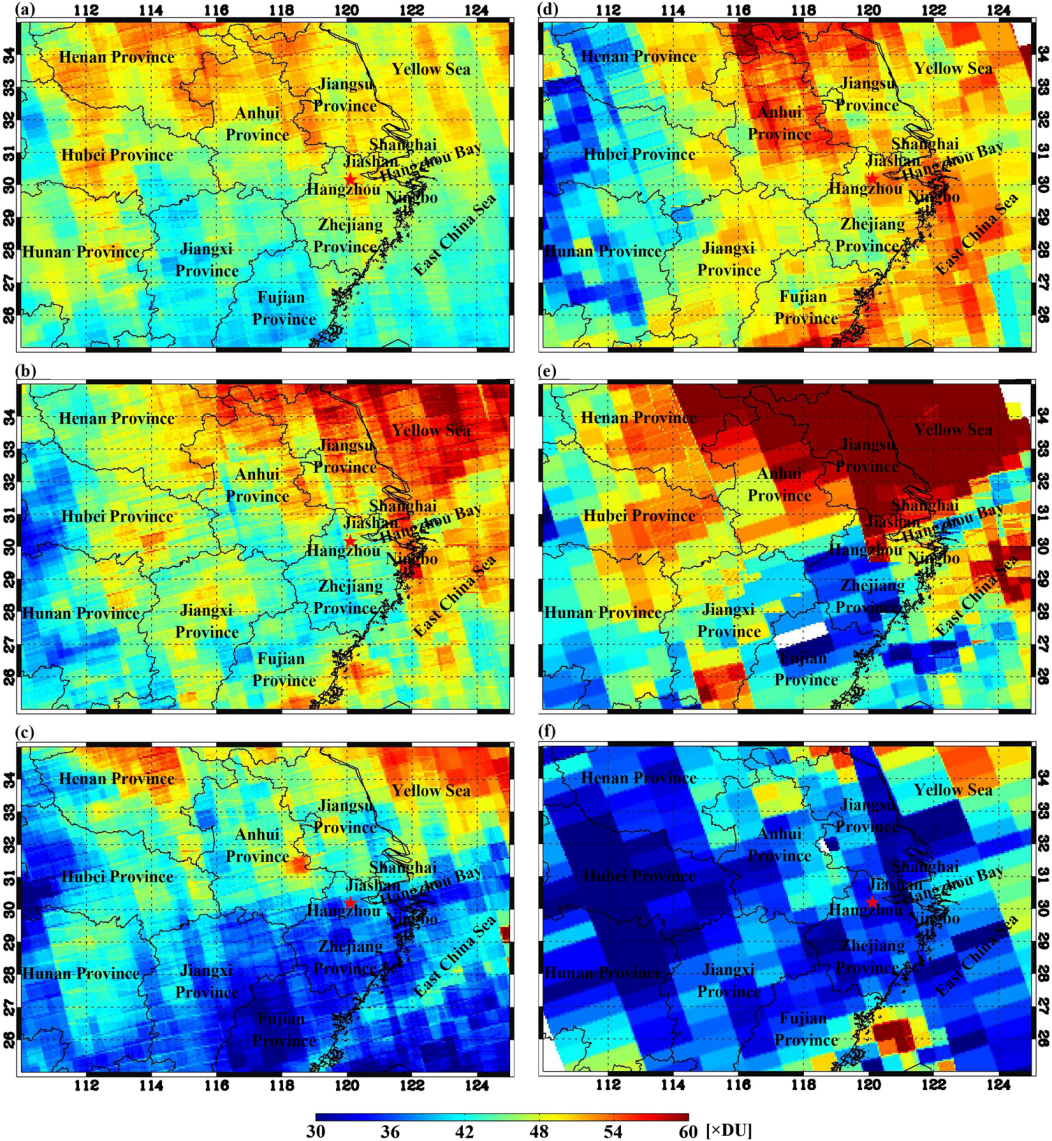
Average VCD Maps of the satellite-derived tropospheric O_3_ during the (**a**) pre-G20 period (Aug. 15–25), (**b**) G20 period (Aug. 26-Sep. 6), (**c**) post-G20 period (Sep. 7–18), (**d**) P1 episode (Aug. 27–31), (**e**) P2 episode (Sep. 2–3) and (**f**) C1 episode (Sep. 5–6). This figure was generated using the IDL 8.2 software (http://www.esrichina.com.cn).

The chemical formation of O_3_ is controlled by NO_x_ or VOCs depending upon which substance is inadequate in the reactions. Accordingly, there are two sensitivity regimes of O_3_ production, namely, the NO_x_-limited and VOC-limited regimes. The satellite-measured ratio of the tropospheric VCD of HCHO to that of NO_2_ has been successfully used to analyze the O_3_ sensitivity in the U.S. and China^22,31–33^. Normally, O_3_ is produced under a VOC-limited regime with a low HCHO/NO_2_ ratio and under a NOx-limited regime with a high HCHO/NO_2_ ratio. Here, we retrieved the tropospheric VCDs of both HCHO and NO_2_ in molecules cm^−2^ and O_3_ in Dobson unit (DU) over Hangzhou and its surrounding regions (Figs 5 and S7,8). The satellite-based O_3_ VCDs during the lidar campaign were well correlated with the average concentrations within 300–2000 m acquired from the lidar observations when the satellite passed over Hangzhou (R = 0.90, P < 0.01, Figure S9). Moreover, it is dramatic that the O_3_ VCDs were also significantly correlated with the DMA-8h O_3_ concentrations in the lower lidar layer (R = 0.74, P < 0.01). This may be due to the fact that high O_3_ concentrations usually occur between 11:00 and 17:00, and thus, the DMA-8h O_3_ concentrations in the lower lidar layer were close to the level when the satellite passed over the monitoring site. Although a satellite passes over a target site only once per day, the O_3_ VCD data can readily reflect the O_3_ pollution conditions in the lower-middle boundary layer.

Because of the high correlation between the satellite- and lidar-measured O_3_, we used lidar data to infer the O_3_ VCDs when satellite data were not available on Aug. 26, Sep. 2 and Sep. 4. Then, the variations in the O_3_ VCDs with the HCHO and NO_2_ VCDs in Hangzhou were analyzed. From Aug. 14 to Sep. 18, the HCHO/NO_2_ ratios varied with a wide range from 1.0 to 9.0. As the absolute values of the HCHO and NO_2_ VCDs were quite different, we normalized the original VCDs in order to compare the sensitivity of O_3_ with the variations in HCHO and NO_2_,

1 $${{\rm{X}}}_{{\rm{nor}}}={\rm{X}}/{{\rm{X}}}_{{\rm{ref}}}$$

where X is the VCD for either HCHO or NO_2_, and X_ref_ is the reference VCD of either HCHO or NO_2_ for normalization. Here, we used the average VCD during the pre-G20 period (Aug. 14–25) as a reference. X_nor_ represents the normalized concentration of either HCHO or NO_2_. Then, the slope of the linear regression analysis for O_3_ versus the normalized HCHO or NO_2_ ratios can represent the change rate of O_3_ when the same fraction of variation in either HCHO or NO_2_ occurred. As presented in Table 1, the slopes of O_3_ versus both HCHO and NO_2_ were positive when the HCHO/NO_2_ ratios were below 4, but the O_3_ slope versus HCHO was much higher than that versus NO_2_, indicating a VOC-limited regime. In particular, when the ratios were below 2, the O_3_ was not sensitive to variations in NO_2_. When the ratios were between 2 and 3, the slope versus HCHO reached a maximum, indicating that diminishing VOCs can represent the best benefit for mitigating O_3_ pollution in this ratio range. When the ratios were between 4 and 6, the slopes versus HCHO and NO_2_ were comparable, indicating a transition regime. Moreover, in this ratio range, the slope versus NO_2_ reached a maximum, and thus, the reduction of NO_2_ can represent the greatest advantage for diminishing tropospheric O_3_. When the HCHO/NO_2_ ratios were above 6, the slope versus NO_2_ exceeded that versus HCHO, indicating a NO_x_-limited regime.

**Table 1:** O_3_ production sensitivity regimes and the corresponding slope and correlation coefficient (R^2^) values for the linear regression analysis for O_3_ versus the normalized HCHO or NO_2_ under different HCHO/NO_2_ ratios.

Regime	HCHO/NO_2_	HCHO		NO_2_	
		Slope	R^2^	Slope	R^2^
VOC-limited	<4	11	0.14	6.0	0.06
	<2	6.7	0.56	0.31	0.01
	2–3	42	0.50	19	0.13
	3–4	8.3	0.16	4.8	0.06
Transition	4~6	25	0.79	29	0.94
NO_x_-limited	>6	10	0.62	19	0.71

In this study, the tropospheric NO_2_ VCDs generally showed a decreasing trend after Aug. 14. With the implementation of pollution control measures, the NO_2_ VCDs decreased more rapidly and reached a trough on Sep. 5 (Figure S10). Then, with the end of the control measures, the NO_2_ VCDs increased after Sep. 7. The mean NO_2_ VCD level during the G20 period was lower than those during both pre- and post-G20 periods (Figures S7). The influence of the air quality policy on tropospheric NO_2_ concentrations was both positive and sensitive. However, the tropospheric HCHO VCDs fluctuated frequently and reached a minimum on Sep. 3. The levels during the G20 period were significantly lower than those during the pre-G20 period. After the emission control stage ended, the HCHO VCDs maintained relatively low levels (Figures S8 and S10). The effect of the air quality policy on HCHO was not as immediate as that on NO_2_. This is probably due to its formation through secondary processes and complex sources from both anthropogenic and natural emissions^34,35^.

From Aug. 14 to Sep. 17, O_3_ generally exhibited a decreasing trend (Figure S10). The average O_3_ VCD during the G20 period was lower than that during the pre-G20 period, but higher than that during the post-G20 period (Fig. 5). During the research period, a decrease in either NO_2_ or HCHO could cause a reduction in O_3_ pollution due to the positive correlations between O_3_ and both NO_2_ and HCHO. However, during the earlier control stage from Aug. 26 to Sep. 4, the O_3_ VCDs fluctuated frequently and exhibited two O_3_ pollution episodes (P1 and P2). During those episodes, most of the HCHO/NO_2_ ratios were below 4, indicating a HCHO-limited regime. If using the average levels of HCHO and NO_2_ during the pre-G20 period (Aug. 14–25) as reference values, the HCHO concentrations during the earlier G20 period (before C1 episode) decreased to 20–82% of the reference value, while the NO_2_ concentrations were 39–128% of the reference value. Under the VOC-limited regime, a reduced concentration of VOCs was not enough to eliminate the O_3_ pollution. During the C1 episode, the HCHO concentrations did not evidently change compared with those during the P1 and P2 episodes; meanwhile, the NO_2_ concentrations greatly decreased to 25–36% of the reference value. Accordingly, the HCHO/NO_2_ ratios became 6.0–9.0, revealing a NO_x_-limited regime. Under this regime, the O_3_ concentration decreased obviously with a decrease in NO_2_. In addition, weaker solar radiation intensities due to precipitation and cloudy weather were probably a more important factor on the reduced O_3_ concentrations during the C1 episode. The average SWDOWN flux in the daytime during the C1 episode was only 313 W m^−2^, which is much lower than that during the pre-G20 period and early G20 period (496 W m^−2^).

It is noteworthy that the O_3_-NO_x_-VOCs sensitivities varied in the vertical direction. Based on the WRF-Chem results, we further investigated the relationship between O_3_ and the normalized HCHO and NO_2_ concentrations in different model layers when the satellite passed over the monitoring site using the same analysis method based on VCDs. The sensitivities always showed a VOC-limited regime under lower HCHO/NO_2_ ratios and a NO_x_-limited or transition regime under higher HCHO/NO_2_ ratios in each model layer (Table S1). Below the 10th model layer (~570–660 m), the sensitivities presented VOC-limited regimes on most days, while NO_x_-limited regimes dominated between the 11th (~660–800 m) and the 18th model layers (~2.6–3.0 km). Furthermore, the slopes of O_3_ versus both HCHO and NO_2_ generally decreased with increasing heights, especially above the 16th model layer (~1.8–2.1 km). This may be caused by relatively stable O_3_ concentrations at higher latitudes, which was supported by the decreasing trend in the relative standard deviation (RSD) of O_3_ with increasing altitude (Table S1), due to extra O_3_ sources such as exchange with the stratosphere in addition to local photochemical production^36^.

### Roles of regional and vertical transports

**Figure 6:**
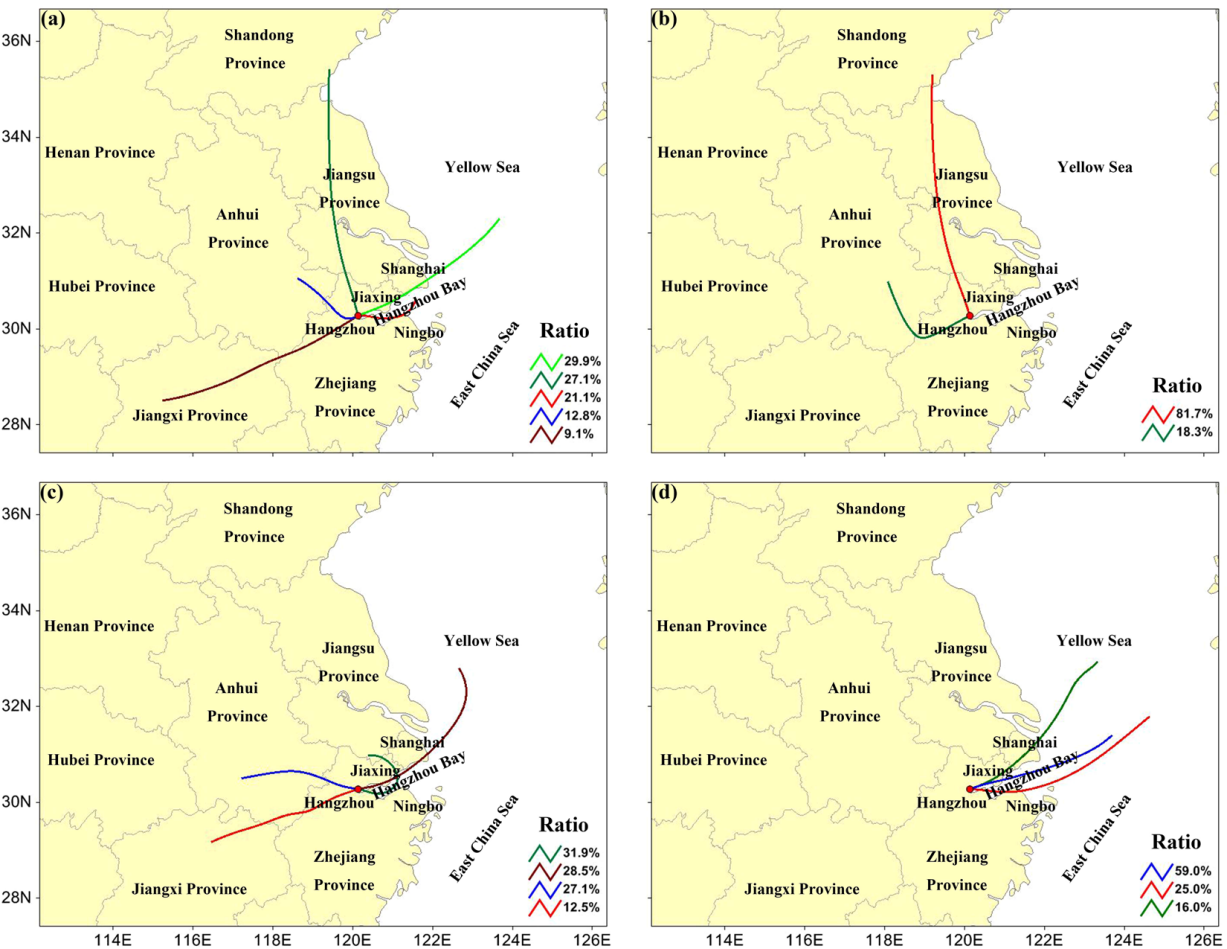
Cluster analysis of the 1-day air mass BTs arriving at the lidar site at 300 m, 400 m and 500 m AGL using meteorological data from the WRF-Chem modelling during (**a**) the whole lidar campaign, (**b**) the P1 episode, (**c**) the P2 episode and (**d**) the C1 episode. The base map was generated using the TrajStat 1.2.2 software (http://www.meteothinker.com).

To determine the influence of regional transport on the O_3_ concentration, 1-day air mass back trajectories (BTs) arriving at the lidar site at the bottom (300 m AGL), middle (400 m AGL) and top (500 m AGL) of the lower lidar layer were calculated for every hour using the Hybrid Single-Particle Lagrangian Integrated Trajectory (HYSPLIT) model. Meteorological data from both the Global Data Assimilation System (GDAS) and the WRF-Chem modelling were adopted to drive the HYSPLIT model. The BTs calculated using the two sets of meteorological data were quite similar, and mainly originated from the east, north and west of the lidar site (Figure S11). Here, we used the BTs calculated using the meteorological data from the WRF-Chem modelling for further discussion and divided the BTs into 5 groups via cluster analysis (Fig. 6a). Owing to the noble diurnal cycle of O_3_, we used the daily averaged O_3_ concentrations instead of the hourly averaged data to compare the O_3_ concentrations with different air masses. The air mass BTs for Cluster 1, accounting for 29.9% of all BTs, originated in the East China Sea and Yellow Sea, and arrived at Hangzhou from the northeast through the cities of Shanghai and Jiaxing. The corresponding daily O_3_ concentrations were relatively low with an average of 48 ± 10 ppbv. The BTs of Cluster 3, accounting for 21.1% of the total, originated from the Hangzhou Bay and arrived at Hangzhou directly over the ocean. The O_3_ concentrations for this BT group were also relatively low (54 ± 10 ppbv). The air masses for Clusters 2, 4 and 5 came from the north, northwest and west of Hangzhou, respectively, and were transported entirely over the continent. The O_3_ concentrations for these air masses were relatively high (72 ± 11, 66 ± 16 and 71 ± 5 ppbv for Clusters 2, 4 and 5, respectively). Particularly, the air mass of Cluster 2 passed through all of Jiangsu Province and the northern part of Zhejiang Province, while the air mass of Cluster 5 passed through western Zhejiang Province and northern Jiangxi Province. Jiangsu and Zhejiang Provinces are two of the most developed provinces in China, and the human activities therein are very intense. Overall, the tropospheric O_3_ concentrations over Jiangsu Province, the adjacent Yellow Sea and the northern part of Zhejiang Province were much higher than those over the other areas (Fig. 5). Therefore, when the air masses originated from the north and northeast of Hangzhou, O_3_ concentrations were usually high. Moreover, the differences in the O_3_ concentrations among the air mass BTs for Clusters 1, 3, 4 and 2/5 were all significant (P < 0.01), indicating that regional transport played a non-negligible role on the O_3_ concentrations in the lower layers over Hangzhou.

Furthermore, cluster analysis was also performed specifically for the P1, P2 and C1 episodes (Fig. 6b–d). During the P1 episode, two clusters were determined with 81.7% of the BTs from the north passing through all of Jiangsu Province and northern Zhejiang Province and 18.3% of the BTs originating from the southwest passing through western Zhejiang Province and southern Anhui Province. During the P2 episode, four clusters were determined with 31.9% of the BTs (for Cluster 1) originating in the city of Jiaxing to the northeast and passing through the Hangzhou Bay before arriving at Hangzhou. The O_3_ concentrations in Jiaxing were much higher than those in Hangzhou during Sep. 4–9 (Figure S12), further confirming the possibility of O_3_ input though these air masses. The air masses for Cluster 3 (27.1%) and Cluster 4 (12.5%) were derived from southern Anhui Province and northern Jiangxi Province, respectively. The other 28.5% of the air mass (for Cluster 2) originated from the East China Sea and Yellow Sea, where the O_3_ was also relatively high (Fig. 5e). However, during the C1 episode, almost all of the BTs originated from the East China Sea and Yellow Sea, where the O_3_ greatly diminished (Fig. 5f). This change in the air masses played an important role on the decrease in the O_3_ during the C1 episode. Low O_3_ concentrations during the C1 episode were also reproduced using the WRF-Chem model without modifying the emission inventory. This indicates that the meteorological conditions rather than the control measures played a dominant role on the O_3_ reduction during the C1 episode.

Following Jiang, *et al*.^26^, the vertical and horizontal transport fluxes were calculated using the simulated wind speed on the grid border multiplied by the O_3_ concentration for the corresponding grid from which the airflow comes. For a certain grid, the input flux is positive and the output flux is negative. The net vertical flux is the algebraic sum of fluxes for the objective grid with its upper and lower grid, while the net horizontal flux is the algebraic sum of fluxes for the objective grid with the 4 grids surrounding it. For the vertical flux calculation, the lidar-measured O_3_ concentrations were adopted; meanwhile, for the horizontal flux calculation, the O_3_ concentrations simulated by WRF-Chem were adopted. At 500 m AGL, the average net flux was 0.02 ppbv m s^−1^ with −0.13 ppbv m s^−1^ output to its upper layer and 0.15 ppbv m s^−1^ input from its lower layer; meanwhile, at 1000 m AGL, the net average flux was 0.052 ppbv m s^−1^ with 0.043 ppbv m s^−1^ input from its higher layer and 0.013 ppbv m s^−1^ input from its lower layer (Figure S13a). Particularly, the vertical fluxes at 500 m suggested weak net input during the P1 episode and weak net output during the P2 episode, and that the transport of O_3_ was entirely directed from the lower layer to the upper layer. At 1000 m, during the P1 episode, the average net flux was 0.12 ppbv m s^−1^ with 0.05 ppbv m s^−1^ input from its upper layer and 0.07 ppbv m s^−1^ input from its lower layer; meanwhile, during the P2 episode, the average net flux was 0.03 ppbv m s^−1^ with 0.13 ppbv m s^−1^ input from the lower layer and 0.10 ppbv m s^−1^ output to the higher layer. Input from the free troposphere to the boundary layer was not observed during either of the two episodes. This is coincident with the conclusion reached in a previous study via ozonesonde measurements and modelling analysis that the influence of stratospheric downward transport is not an important source of O_3_ in the lower troposphere^36^. However, the horizontal fluxes were much higher than the vertical horizontal fluxes (Figure S13b). In addition, as discussed above, the O_3_ concentrations showed great difference with different air mass BTs. Compared with vertical exchange, regional transport played a much more important role on O_3_ in the boundary layer.

## Methods

### Ozone Lidar

The O_3_ profiles in the lower troposphere were monitored using differential absorption lidar (DIAL) technology in urban Hangzhou from Aug. 25 to Sep. 9. The lidar detected the absorption of laser light at three wavelengths of 266 nm, 289 nm and 316 nm. The typical laser pulse energy was approximately 90 mJ with a frequency of 10 Hz. The laser beam was emitted with a divergence of 0.3 milliradian (mrad) and with a field of view of 0.5 mrad, causing an overlap height of approximately 300 m. The laser pulse at 266 nm was created using a Nd:YAG medium and the two other lasers pulses at 289 nm and 316 nm were produced by sending the 266 nm beam through a Raman tube filled with D_2_ and H_2_. Then, these three laser pulses at 266 nm, 289 nm and 316 nm passed through the open atmosphere and underwent extinction due to scattering by aerosols and air molecules and absorption by trace gases. Finally, the backscatter signals of those three wavelengths were collected by a telescope. The ozone profiles were obtained using DIAL retrieval algorithms, which were described in detail by Fan, *et al*.^37^. The vertical profiles of aerosol extinction coefficients at 316 nm were retrieved using the Fernald inversion method^38^. The lidar observation time resolution was approximately 12 min, and the original vertical resolution was 7.5 m. To improve the signal to noise ratio, the reported data were subjected to 10-point smoothing in the vertical direction. The O_3_ concentrations measured using this lidar were compared with a series of simultaneous balloon-based measurements acquired during our previous study, and the results from the two methods showed good agreement^39^. The error budget for the O_3_ data was calculated as the sum of a statistical error, a systematic error related to aerosol scattering and a systematic error related to aerosol extinction following Papayannis, *et al*.^40^. The error budget profile of the retrieved O_3_ concentrations with a vertical resolution of 75 m is presented in Figure S1. Only the O_3_ concentrations with relative errors below 20% were adopted for further analysis in this study.

### USTC’S OMI product

The VCDs of O_3_, NO_2_ and HCHO in the troposphere were retrieved based on OMI satellite products, which have been widely used in previous studies^22,41–43^. The OMI sensor mounted on the NASA Earth Observation System (EOS) Aura satellite provides measurements of ultraviolet/visible (UV/VIS) nadir solar backscattering from the Earth’s atmosphere and the surface. The Aura satellite was launched on July 15, 2004, with a high spatial resolution of 13 km × 24 km^44^. Aura follows a near-polar, sun-synchronous, 705-km-altitude orbit with a local ascending equator-crossing time of 13:30^44^. In this study, we retrieved the USTC’s OMI product for trace gases, which was developed based on OMI’s primary product and has proven to be more suitable for the atmospheric conditions in China^22^. For NO_2_, we used the OMI Level 1B VIS Global Radiances Data product (OML1BRVG) (https://disc.gsfc.nasa.gov/Aura/data-holdings/OMI/oml1brvg_v003.shtml) to retrieve the NO_2_ slant column density (SCD) by nonlinear least squares method^45^. Then, the vertical profiles of NO_2_ profiles from the WRF-Chem modelling were extracted to calculate the air mass factor (AMF). Finally, the NO_2_ VCD was determined using the AMF:

2 $${\rm{VCD}}={\rm{SCD}}/{\rm{AMF}}$$

Similarly, for HCHO, we adopted the HCHO SCD included in the Level-2 OMI Formaldehyde Data product (OMHCHO V003) (https://disc.gsfc.nasa.gov/Aura/data-holdings/OMI/omhcho_v003.shtml) and the HCHO profiles from the WRF-Chem modelling to calculate the HCHO VCD. For O_3_, we used the vertical OMI/Aura O_3_ profile product, which includes the tropospheric O_3_ column (https://avdc.gsfc.nasa.gov/pub/data/satellite/Aura/OMI/V03/L2/OMPROFOZ/).

### WRF-Chem modelling

The WRF-Chem model (version 3.7) was used to simulate the air pollutants and meteorological parameters from Aug. 25 to Sep. 9, 2016. The configuration of the modelling was described in detail in our previous study^22^. In Brief, the model domain was centred at 35.0° N, 110.0°E; it encompassed East China and its surrounding areas, with a grid resolution of 20 × 20 km and 26 vertical layers from the ground level to the height with a pressure of 50 hPa. The National Centers for Environmental Prediction (NCEP) 6-hour final operational global (FNL) data with a spatial resolution of 1° × 1° were used to provide the initial and boundary conditions of the meteorological field for simulation. To reproduce the meteorology more effectively, the NCEP’s ADP global upper-air observations (NCAR archive ds351.0) were assimilated every 6 hours. The key physical parameterization options for this modelling scheme included the Noah land surface for land-atmosphere interactions, the Lin microphysics scheme with the Grell cumulus parameterization for cloud and precipitation processes, the YSU boundary layer scheme and the RRTMG short- and long-wave radiation scheme. As presented in Fig. S14 (a), the modelled vertical profiles of the main meteorological parameters, such as the temperature, pressure, water vapor mixing ratio and wind speed/direction, effectively reproduced the results observed via radiosondes (http://weather.uwyo.edu/). The CBMZ (Carbon-Bond Mechanism version Z) photochemical mechanism combined with the MOSAIC (Model for Simulating Aerosol Interactions and Chemistry) aerosol model was used to simulate the chemical process in the atmosphere. The Multi-resolution Emission Inventory for China (MEIC, http://www.meicmodel.org/)^46,47^ was obtained to provides anthropogenic emissions. The biogenic emissions were calculated online using the Model of Emissions of Gases and Aerosols from Nature (MEGAN) embedded in the WRF-Chem model.

### Air mass BTs and cluster analysis

One-day air mass BTs arriving at the lidar site at 300, 400 and 500 m AGL were analyzed every hour using the HYSPLIT model (http://ready.arl.noaa.gov/HYSPLIT.php) from the National Oceanic and Atmospheric Administration (NOAA). The meteorological data from both the GDAS (https://www.ncdc.noaa.gov/data-access/model-data/model-datasets/global-data-assimilation-system-gdas) and the WRF-Chem modelling were adopted to drive the HYSPLIT model. Then, the calculated air mass BTs were classified into several groups through cluster analysis. Cluster analysis is a multivariate statistical technique that assigns a large amount of members (trajectories) to a given group (cluster) by maximizing the external variability among the different groups and minimizing the internal variability within each group based on the trajectory coordinates^48,49^.

## Electronic supplementary material

Supplementary Information
