# Supplementary material for: Characterization of ozone in the lower troposphere during the 2016 G20 conference in Hangzhou
Source: Sci Rep. 2017 Dec 12;7:17368. doi: 10.1038/s41598-017-17646-x (PMC5727200; doi:10.1038/s41598-017-17646-x)
Supplement: Supplementary file 1 — Supplementary Information [file 41598_2017_17646_MOESM1_ESM.pdf]

Supplementary information for the manuscript:

Characterization of ozone in the lower troposphere during the 2016 G20 conference in Hangzhou

Wenjing Su[1], Cheng Liu[1, 2, 3], Qihou Hu[2], Guangqiang Fan[2], Zhouqing Xie[1, 2, 3], Xin Huang[4], Tianshu Zhang[2], Zhenyi Chen[2], Yunsheng Dong[2], Xiangguang Ji[1,2], Haoran Liu[1], Zhuang Wang[1,2], Jianguo Liu[2, 3]

1. School of Earth and Space Sciences, University of Science and Technology of China, Hefei, 230026, China

2. Key Lab of Environmental Optics & Technology, Anhui Institute of Optics and Fine Mechanics, Chinese Academy of Sciences, Hefei, 230031, China

3. Center for Excellence in Regional Atmospheric Environment, Institute of Urban Environment, Chinese Academy of Sciences, Xiamen, 361021, China

4. Institute for Climate and Global Change Research & School of Atmospheric Sciences, Nanjing University, Nanjing, 210023, China

Correspondence and requests for materials should be addressed to C.L. (chliu81@ustc.edu.cn) or Q.H. (qhhu@aiofm.ac.cn) or G.F. (gqfan@aiofm.ac.cn)

To complement the information given in the main manuscript, the following sections provide supporting information: Table S1 and Figures S1-S14.

Table S1. O_3 production sensitivity regimes, the corresponding number of days and the corresponding slope and correlation coefficient (R^2) values for the linear regression analysis for O_3 versus the normalized HCHO or NO_2 under different HCHO/NO_2 ratios, in addition to the relative standard deviation (RSD) of O_3 during the research period (Aug. 14-Sep. 18) in respective model layers when the satellite passed over our monitoring site. The O_3, HCHO and NO_2 concentrations in ppbv were simulated using the WRF-Chem model.

Model layer	RSD of O_3	Regime	Number of days	HCHO/NO_2	HCHO		NO_2	
					Slope	R^2	Slope	R^2
1st (~0-20 m)	48%	VOC-limited	28	<0.6	66	0.26	-29	0.24
		Transition	4	0.6-0.7	86	0.57	97	0.33
		NO_x-limited	4	>0.7	153	0.86	221	0.99
2nd (~20-60 m)	47%	VOC-limited	25	<0.5	73	0.32	-27	0.20
		Transition	7	0.5-0.7	99	0.76	119	0.38
		NO_x-limited	4	>0.7	157	0.85	224	0.99
3rd (~60-110 m)	46%	VOC-limited	28	<0.6	61	0.24	-24	0.21
		Transition	4	0.6-0.7	90	0.60	89	0.32
		NO_x-limited	4	>0.7	158	0.85	225	0.99
4th (~110-150 m)	45%	VOC-limited	24	<0.5	71	0.32	-26	0.21
		Transition	7	0.5-0.7	50	0.10	40	0.02
		NO_x-limited	5	>0.7	132	0.82	236	0.95
5th (~150-200 m)	44%	VOC-limited	23	<0.5	65	0.32	-22	0.16
		Transition	9	0.5-0.8	74	0.30	81	0.29
		NO_x-limited	4	>0.8	161	0.84	226	0.99
6th (~200-280 m)	43%	VOC-limited	23	<0.5	62	0.33	-18	0.11
		Transition	9	0.5-0.8	82	0.37	83	0.32
		NO_x-limited	4	>0.8	163	0.84	228	0.99
7th (~280-390 m)	41%	VOC-limited	23	<0.5	59	0.32	-14	0.07
		Transition	9	0.5-0.8	96	0.51	94	0.43
		NO_x-limited	4	>0.8	165	0.83	230	0.99
8th (~390-480 m)	40%	VOC-limited	23	<0.5	56	0.32	-8.7	0.03
		Transition	7	0.5-0.8	107	0.87	106	0.67
		NO_x-limited	6	>0.8	150	0.75	237	0.63
9th (~480-570 m)	38%	VOC-limited	14	<0.4	51	0.43	6.9	0.03
		Transition	13	0.4-0.7	55	0.49	40	0.36
		NO_x-limited	9	>0.7	140	0.79	200	0.65
10th (~570-660 m)	36%	VOC-limited	22	<0.5	53	0.38	7.8	0.02
		Transition	7	0.5-0.8	113	0.90	129	0.75
		NO_x-limited	7	>0.8	134	0.88	270	0.93
11th (~660-800 m)	35%	VOC-limited	8	<0.4	33	0.23	8.9	0.07
		Transition	13	0.4-0.7	45	0.44	34	0.42
		NO_x-limited	15	>0.7	120	0.90	174	0.79

Table S1. Continued.

Model layer	RSD of O_3	Regime	Number of days	$HCHO/NO_2$	HCHO		NO_2	
12th (~800-1000 m)	34%	VOC-limited	8	<0.5	62	0.41	-37	0.21
		Transition	8	0.5-0.7	60	0.39	65	0.47
		NO_x-limited	20	>0.7	101	0.82	145	0.70
13th (~1.0-1.3 km)	33%	VOC-limited	14	<1	31	0.21	-3.6	0.06
		Transition	6	1-1.5	54	0.27	65	0.16
		NO_x-limited	16	>1.5	31	0.42	62	0.49
14th (~1.3-1.5 km)	35%	VOC-limited	12	<2	8.5	0.03	3.9	0.05
		Transition	12	2~3	23	0.22	22	0.23
		NO_x-limited	12	>3	19	0.33	38	0.42
15th (~1.5-1.8 km)	36%	Transition	24	<4	6.7	0.01	5.1	0.04
		NO_x-limited	12	>4	42	0.37	60	0.35
16th (~1.8-2.1 km)	38%	VOC-limited	17	<4	8.5	0.01	4.2	0.06
		NO_x-limited	19	>4	39	0.31	50	0.46
17th (~2.1-2.6 km)	38%	VOC-limited	12	<5	25	0.12	5.0	0.16
		NO_x-limited	24	>5	32	0.37	43	0.74
18th (~2.6-3.0 km)	32%	VOC-limited	11	<6	46	0.31	17	0.79
		NO_x-limited	25	>6	22	0.37	29	0.68
19th (~3.0-3.3 km)	26%	VOC-limited	19	<8	34	0.47	13	0.94
		Transition	12	8~12	12	0.20	13	0.10
		NO_x-limited	5	>12	-13	0.04	31	0.40
20th (~3.3-3.7 km)	25%	VOC-limited	29	<15	25	0.48	8.8	0.66
		NO_x-limited	7	>15	59	0.47	89	0.90
21th (~3.7-4.0 km)	24%	VOC-limited	24	<10	15	0.28	0.81	0.18
		Transition	6	10~15	29	0.07	22	0.02
		NO_x-limited	6	>15	-40	0.45	33	0.57
22th (~4.0-4.9 km)	25%	VOC-limited	22	<10	6.3	0.11	0.26	0.14
		Transition	9	10~15	37	0.30	41	0.11
		NO_x-limited	5	>15	11	0.04	82	0.72
23th (~4.9-6.2 km)	30%	VOC-limited	10	<8	13	0.12	4.0	0.28
		Transition	19	8~15	52	0.47	46	0.41
		NO_x-limited	7	>15	-10	0.34	10	0.08
24th (~6.2-8.0 km)	33%	Transition	5	<4	-24	0.79	-15	0.75
		NO_x-limited	31	>4	30	0.08	61	0.76
25th (~8.0-10 km)	27%	VOC-limited	21	<4	55	0.17	35	0.51
		Transition	6	4~5	-17	0.69	-25	0.66
		NO_x-limited	9	>5	-14	0.02	71	0.36
26th (~10-16 km)	12%	VOC-limited	9	<1	88	0.92	55	0.84
		Transition	21	1~2	23	0.26	45	0.77
		NO_x-limited	6	>2	-7.8	0.09	54	0.71

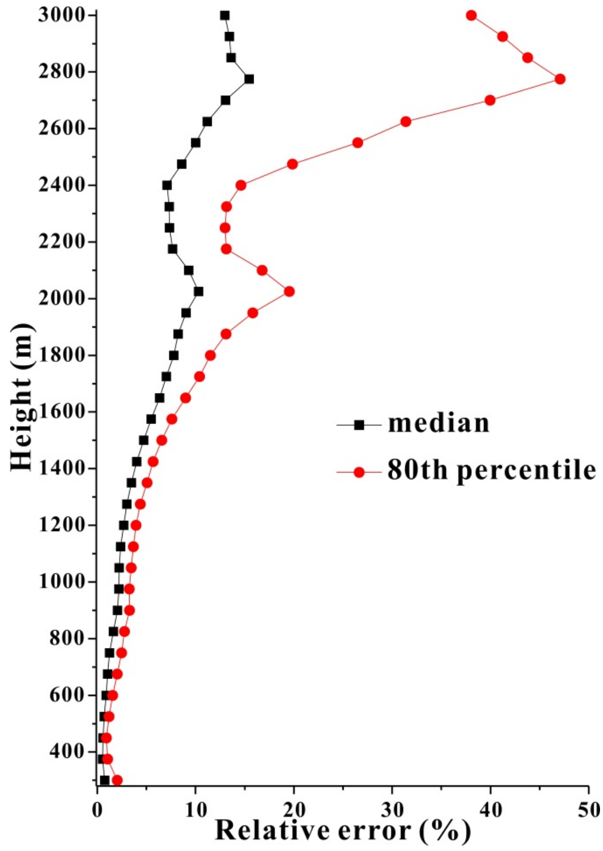

Figure S1. Vertical profiles of the median and 80th percentile of retrieval error budge for O_3 from the lidar observation.

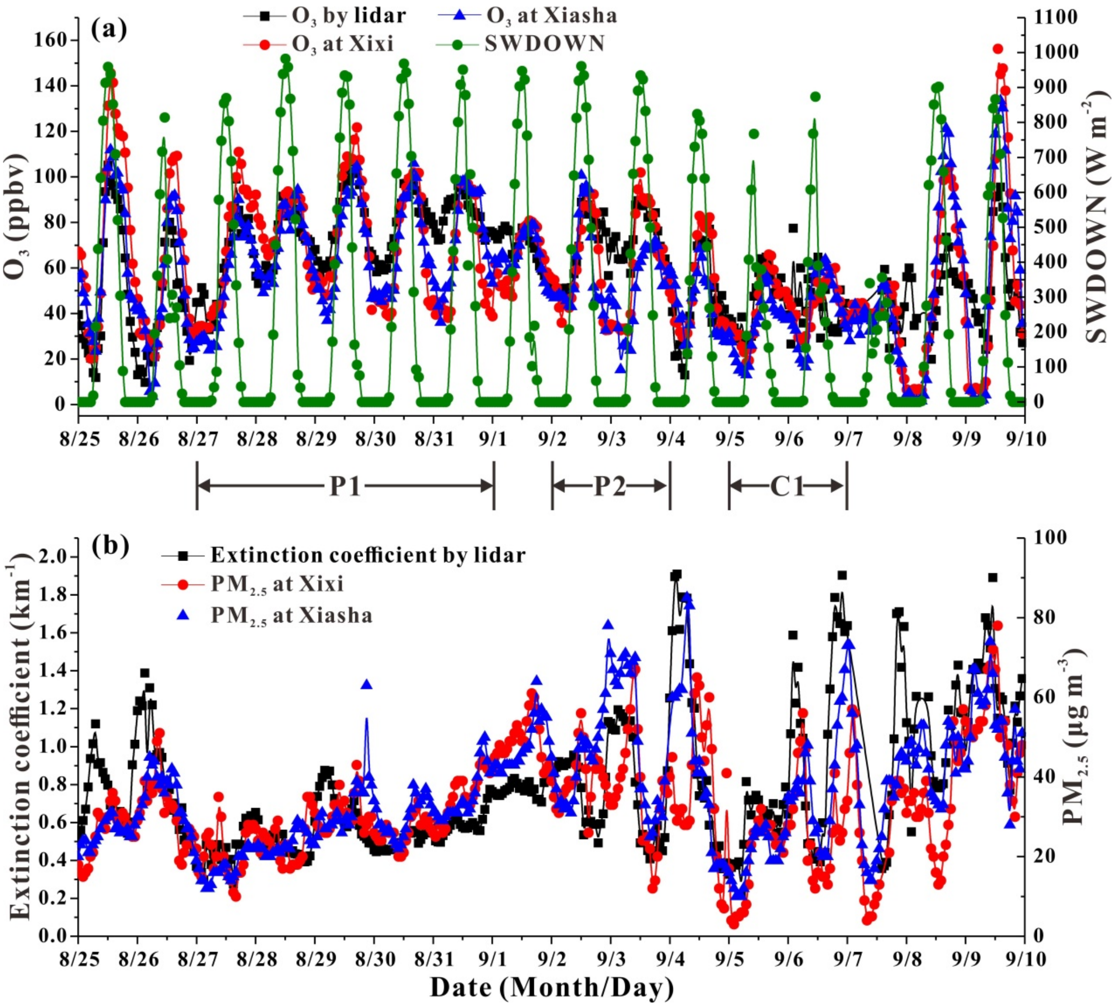

Figure S2. (a) Time series of the measured O_3 concentrations in the lower lidar layer at the lidar site and the surface O_3 concentrations at Xixi and Xiasha, in addition to the downward shortwave radiation (SWDOWN) flux at the ground surface simulated using the WRF-Chem model. (b) Time series of the measured aerosol extinction coefficients in the lower lidar layer at the lidar site and the surface $PM_{2.5}$ concentrations at Xixi and Xiasha. A label on the x-axis of "8/26" represents a time of 00:00:00 on Aug. 26 (local time, UTC+8), and the same for Figures S3, S10, S12 and S13.

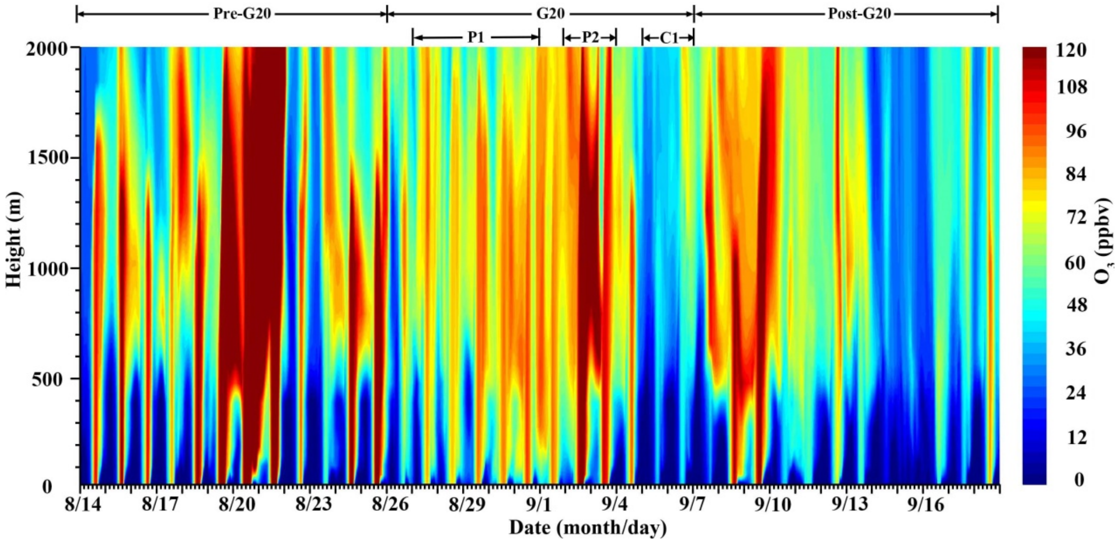

Figure S3. Time series of the profiles for the O_3 concentration at the lidar site in Hangzhou simulated using the WRF-Chem model.

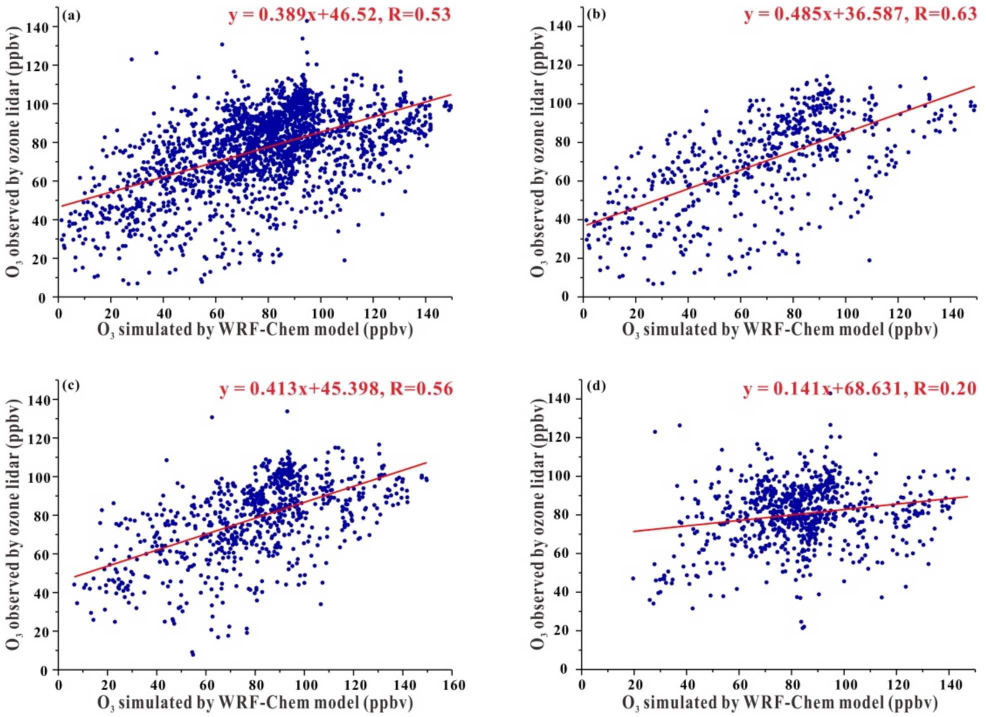

Figure S4. Relationships between the O_3 concentrations simulated using the WRF-Chem model and those measured by the ozone lidar after interpolated into the model layers for (a) the whole lidar layers (300-2000 m), (b) the lower lidar layer, (c) the middle lidar layer and (d) the upper lidar layer.

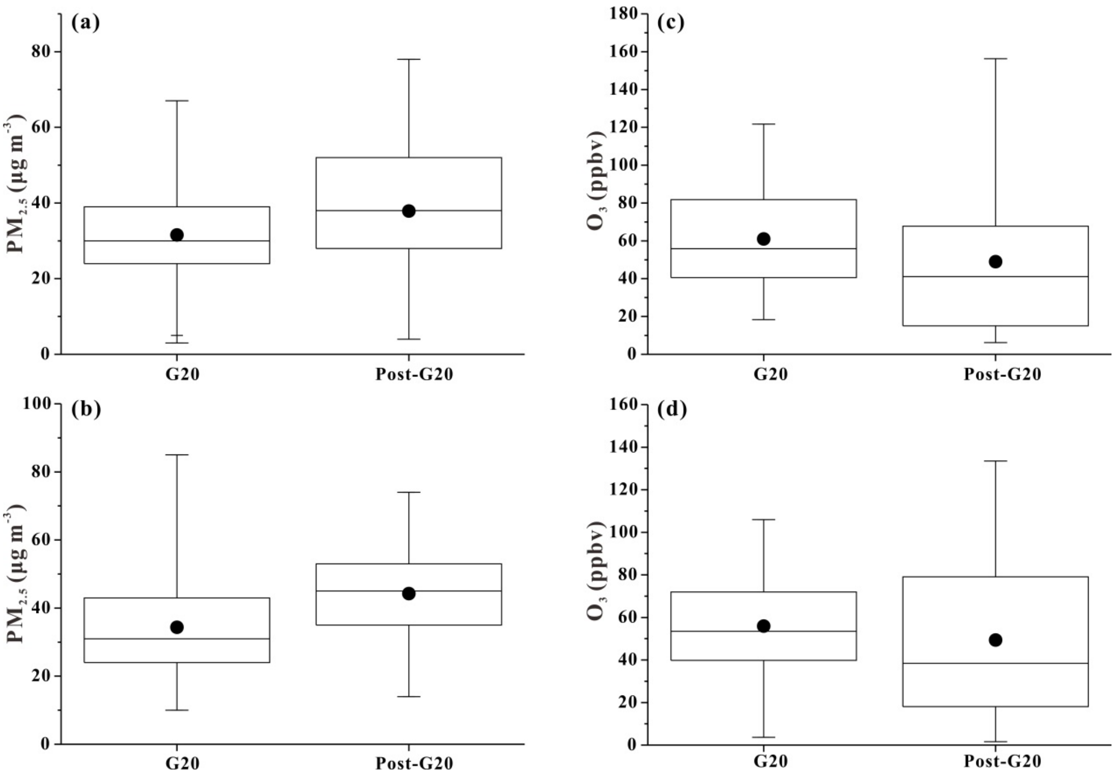

Figure S5. Box-and-whisker plots of the surface $PM_{2.5}$ concentrations during the G20 (Aug. 26-Sep. 6) and post-G20 (Sep. 7-9) periods at (a) Xixi and (b) Xiasha; box-and-whisker plots of the surface O_3 concentrations during the G20 and post-G20 periods at (c) Xixi and (d) Xiasha. The lower and upper boundaries of the boxes represent the 25th and the 75th percentiles, respectively; the whiskers below and above the boxes indicate the minimum and maximum, respectively. The line within the box marks the median; while the dot represents the mean.

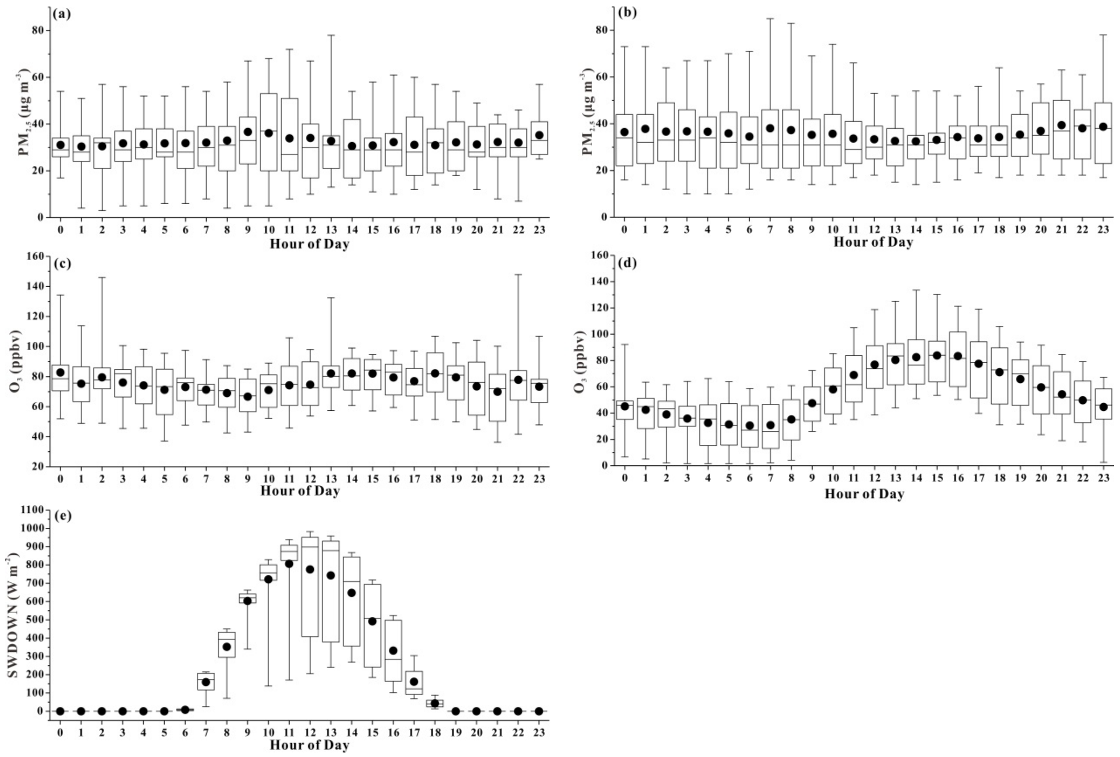

Figure S6. Diurnal variation box-and-whisker plots of the surface $PM_{2.5}$ concentrations at (a) Xixi and (b) Xiasha; diurnal variation box-and-whisker plots of the surface O_3 concentrations at (c) Xixi and (d) Xiasha; (e) diurnal variation box-and-whisker plot of the SWDOWN flux at the lidar site.

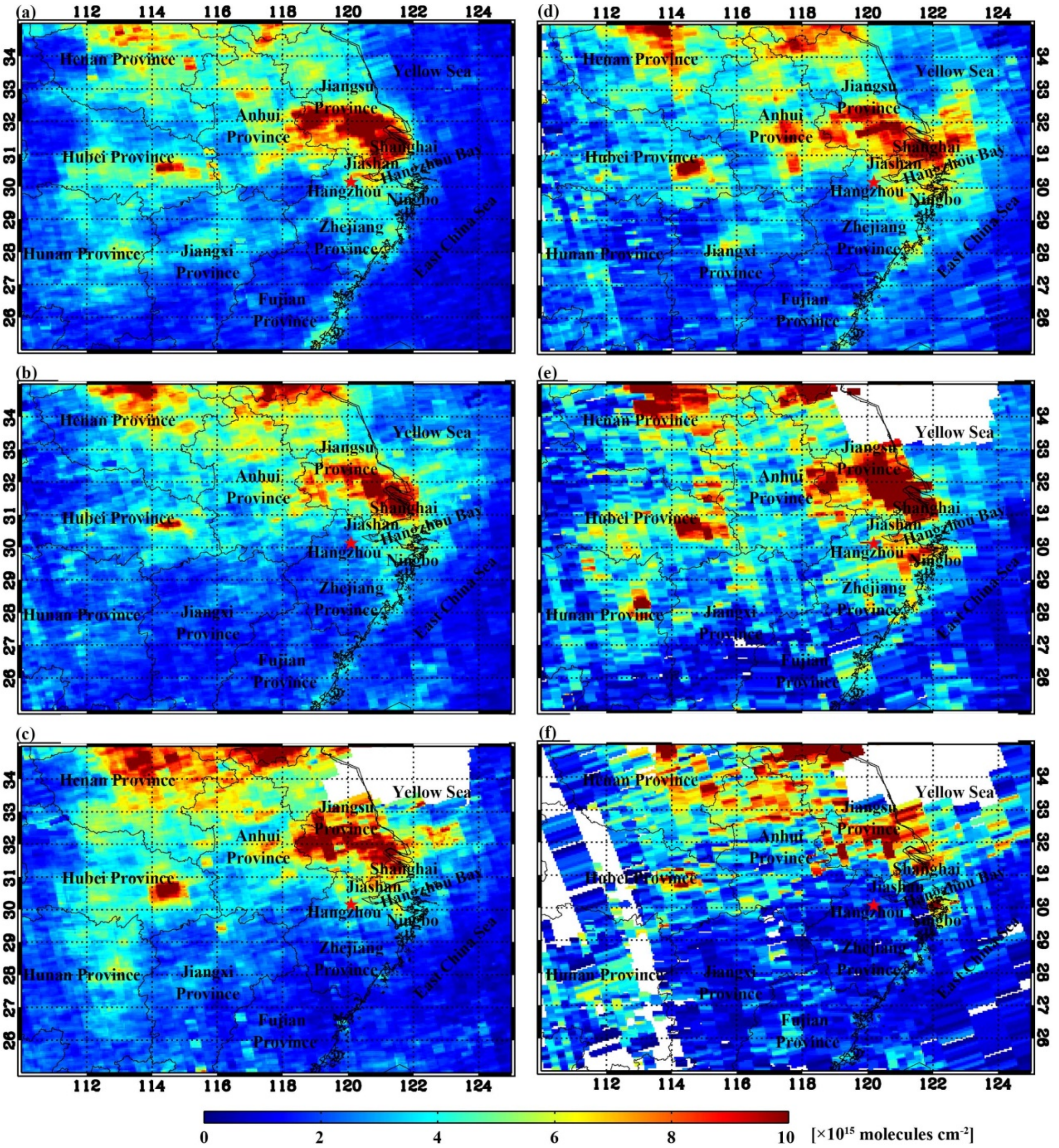

Figure S7. Average VCD Maps of the satellite-derived tropospheric NO_2 during the (a) pre-G20 period (Aug. 14-25), (b) G20 period (Aug. 26-Sep. 6), (c) post-G20 period (Sep. 7-18), (d) P1 episode (Aug. 27-31), (e) P2 episode (Sep. 2-3) and (f) C1 episode (Sep. 5-6). This figure was generated using the IDL 8.2 software (http://www.esrichina.com.cn)

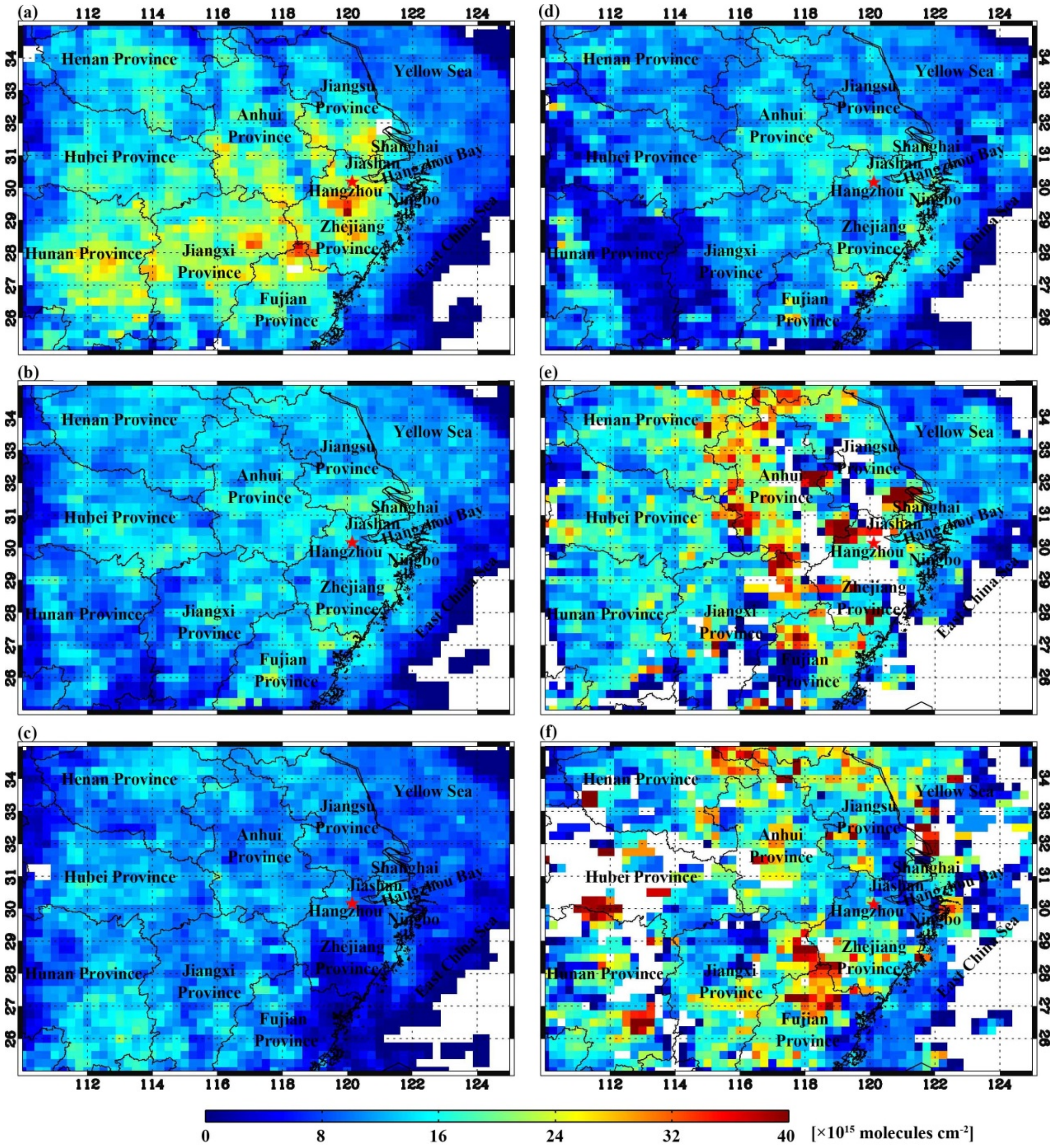

Figure S8. Average VCD Maps of the satellite-derived tropospheric HCHO during the (a) pre-G20 period (Aug. 14-25), (b) G20 period (Aug. 26-Sep. 6), (c) post-G20 period (Sep. 7-18), (d) P1 episode (Aug. 27-31), (e) P2 episode (Sep. 2-3) and (f) C1 episode (Sep. 5-6). This figure was generated using the IDL 8.2 software (http://www.esrichina.com.cn).

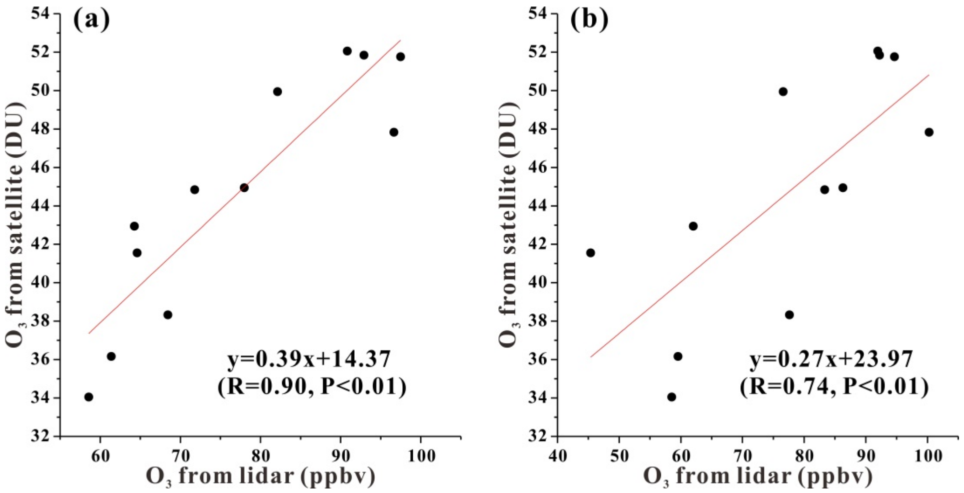

Figure S9. (a) Relationships between the satellite-derived VCDs of the tropospheric O_3 and the averages of the O_3 mixing ratio in the whole lidar layer (300-2000 m AGL) measured by the ozone lidar when the satellite passed over the lidar site. (b) Relationships between the satellite-derived VCDs of the tropospheric O_3 and the O_3 concentrations in the highest daily maximum 8-hour averages (DMA-8h) in the lower lidar layer (300-500 m AGL).

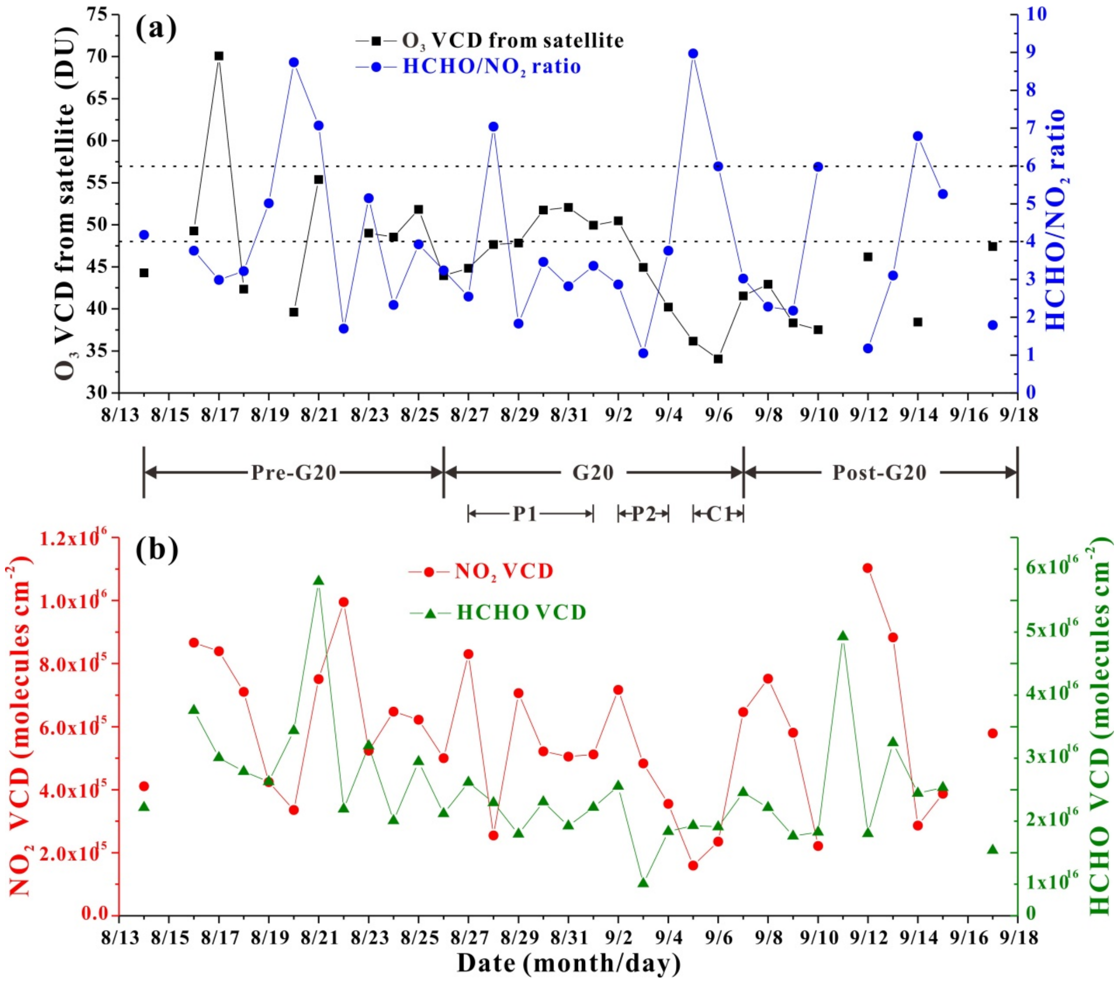

Figure S10. (a) Time series of the VCDs for the tropospheric O_3 and the $HCHO/NO_2$ ratios; (b) Time series of the VCDs for the tropospheric HCHO and NO_2.

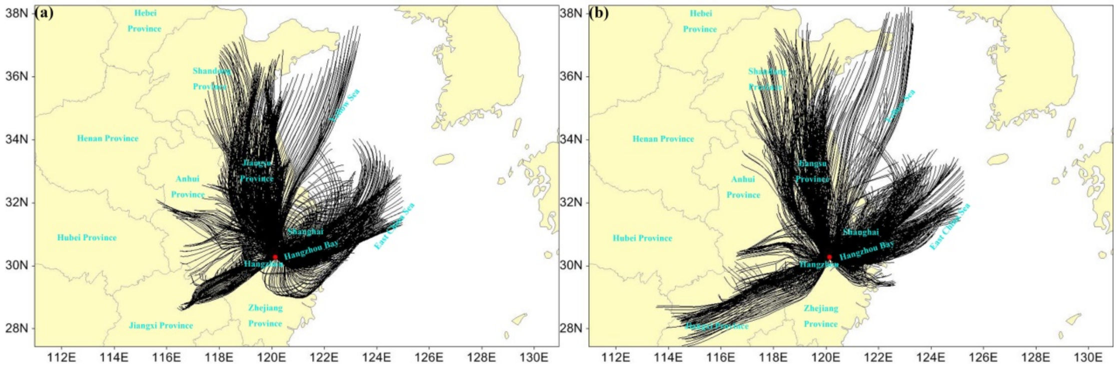

Figure S11. One-day air mass back trajectories (BTs) arriving at the lidar site at 300 m, 400 m and 500 m AGL during the lidar campaign calculated using meteorological data from (a) the Global Data Assimilation System (GDAS) and (b) the WRF-Chem modelling, respectively. The base map was generated using the TrajStat 1.2.2 software (http://www.meteothinker.com).

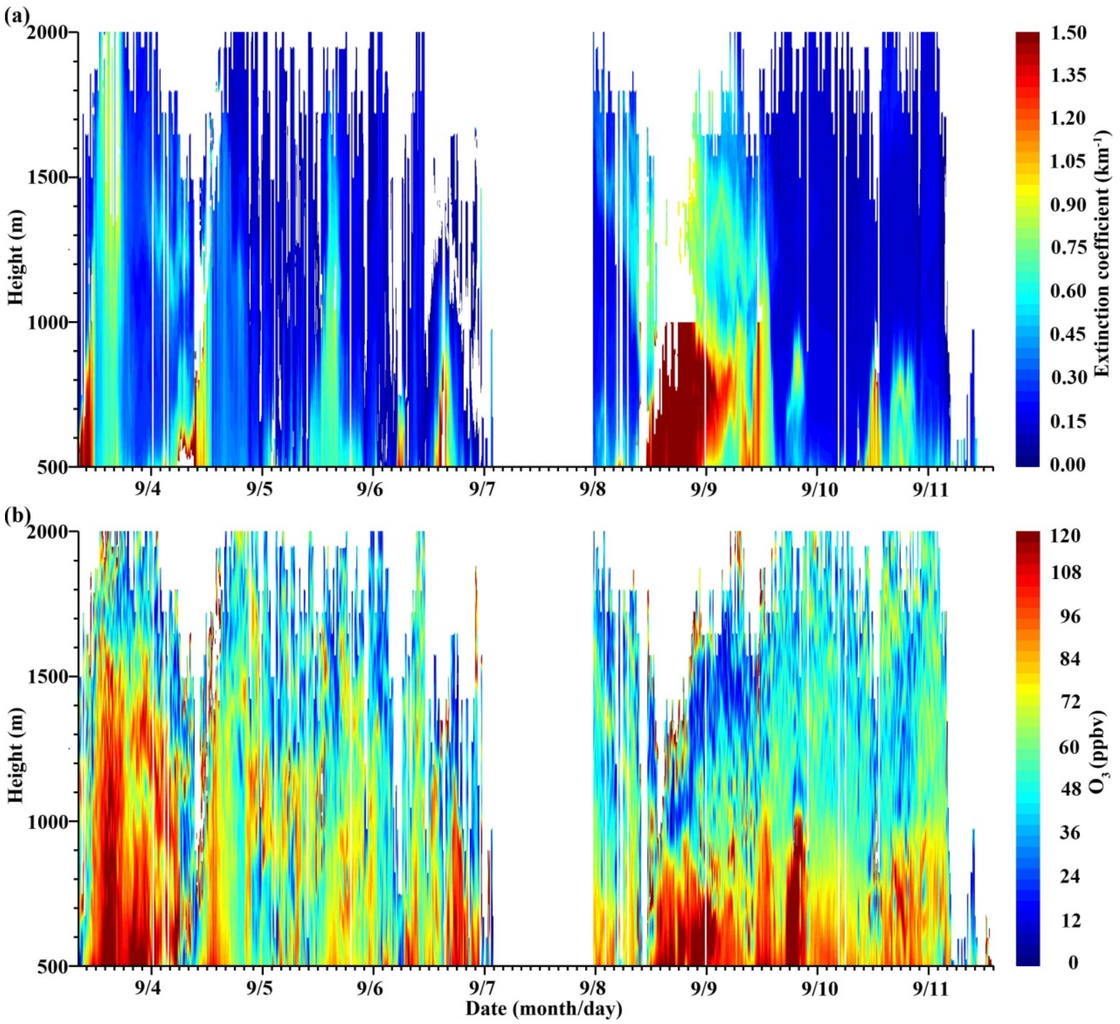

Figure S12. Time series of the vertical profiles for the (a) aerosol extinction coefficient and (b) O_3 concentration measured by the ozone lidar in Jiaxing (30.83° N, 120.87° E).

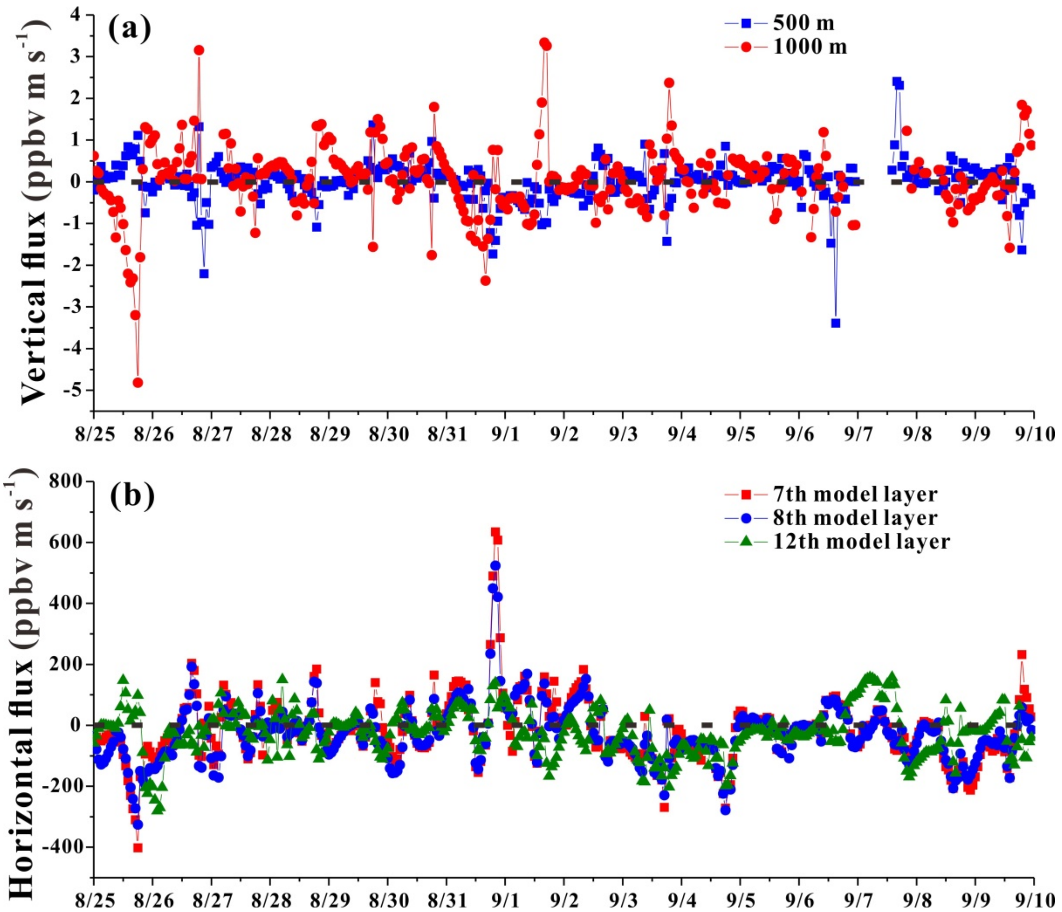

Figure S13. (a) Net vertical fluxes of O_3 at 500 and 1000 m AGL; (b) net horizontal fluxes of O_3 in the 7th (~280-390 m), 8th (~390-480 m) and 12th (~800-1000 m) model layers.

(a)

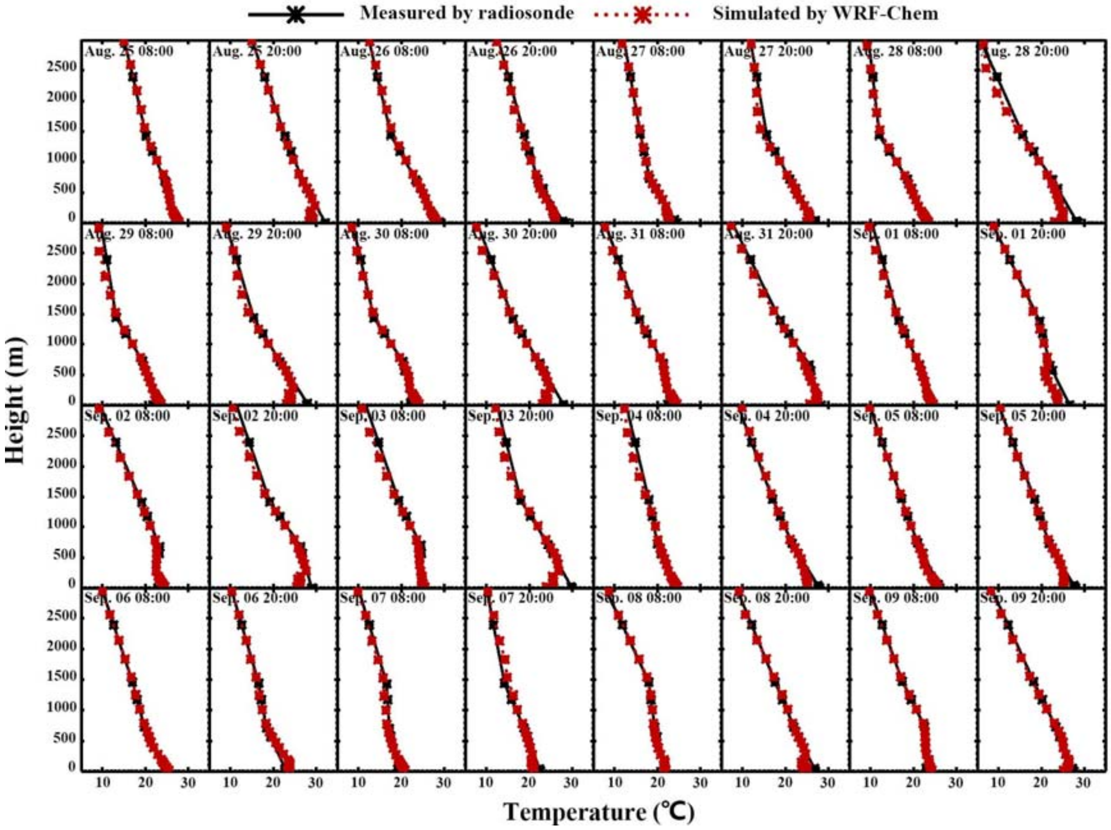
Measured by radiosonde
Simulated by WRF-Chem
Aug. 25 08:00
Aug. 25 20:00
Aug. 26 08:00
Aug. 26 20:00
Aug. 27 08:00
Aug. 27 20:00
Aug. 28 08:00
Aug. 28 20:00
Aug. 29 08:00
Aug. 29 20:00
Aug. 30 08:00
Aug. 30 20:00
Aug. 31 08:00
Aug. 31 20:00
Sep. 01 08:00
Sep. 01 20:00
Sep. 02 08:00
Sep. 02 20:00
Sep. 03 08:00
Sep. 03 20:00
Sep. 04 08:00
Sep. 04 20:00
Sep. 05 08:00
Sep. 05 20:00
Sep. 06 08:00
Sep. 06 20:00
Sep. 07 08:00
Sep. 07 20:00
Sep. 08 08:00
Sep. 08 20:00
Sep. 09 08:00
Sep. 09 20:00
Height (m)
Temperature (℃)

(b)

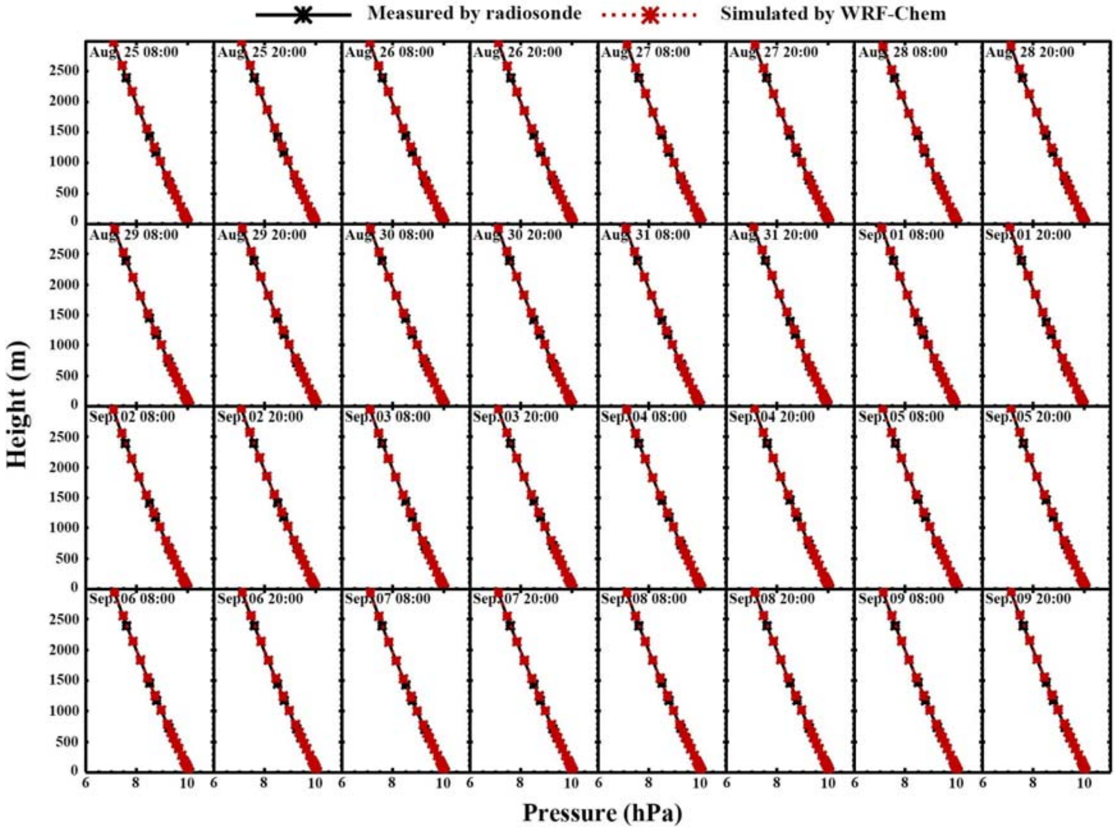
Measured by radiosonde
Simulated by WRF-Chem
Aug. 25 08:00
Aug. 25 20:00
Aug. 26 08:00
Aug. 26 20:00
Aug. 27 08:00
Aug. 27 20:00
Aug. 28 08:00
Aug. 28 20:00
Aug. 29 08:00
Aug. 29 20:00
Aug. 30 08:00
Aug. 30 20:00
Aug. 31 08:00
Aug. 31 20:00
Sep. 01 08:00
Sep. 01 20:00
Sep. 02 08:00
Sep. 02 20:00
Sep. 03 08:00
Sep. 03 20:00
Sep. 04 08:00
Sep. 04 20:00
Sep. 05 08:00
Sep. 05 20:00
Sep. 06 08:00
Sep. 06 20:00
Sep. 07 08:00
Sep. 07 20:00
Sep. 08 08:00
Sep. 08 20:00
Sep. 09 08:00
Sep. 09 20:00
Height (m)
Pressure (hPa)

(c)

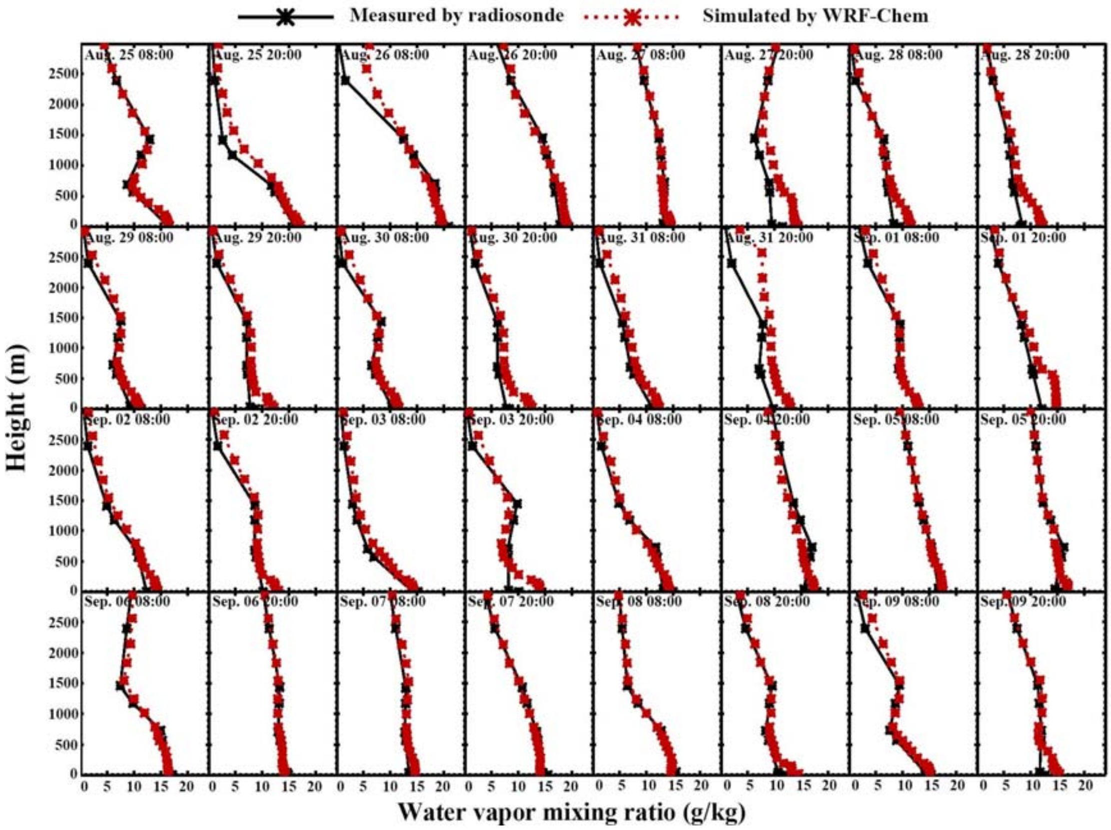
Measured by radiosonde
Simulated by WRF-Chem
Height (m)
Water vapor mixing ratio (g/kg)

(d)

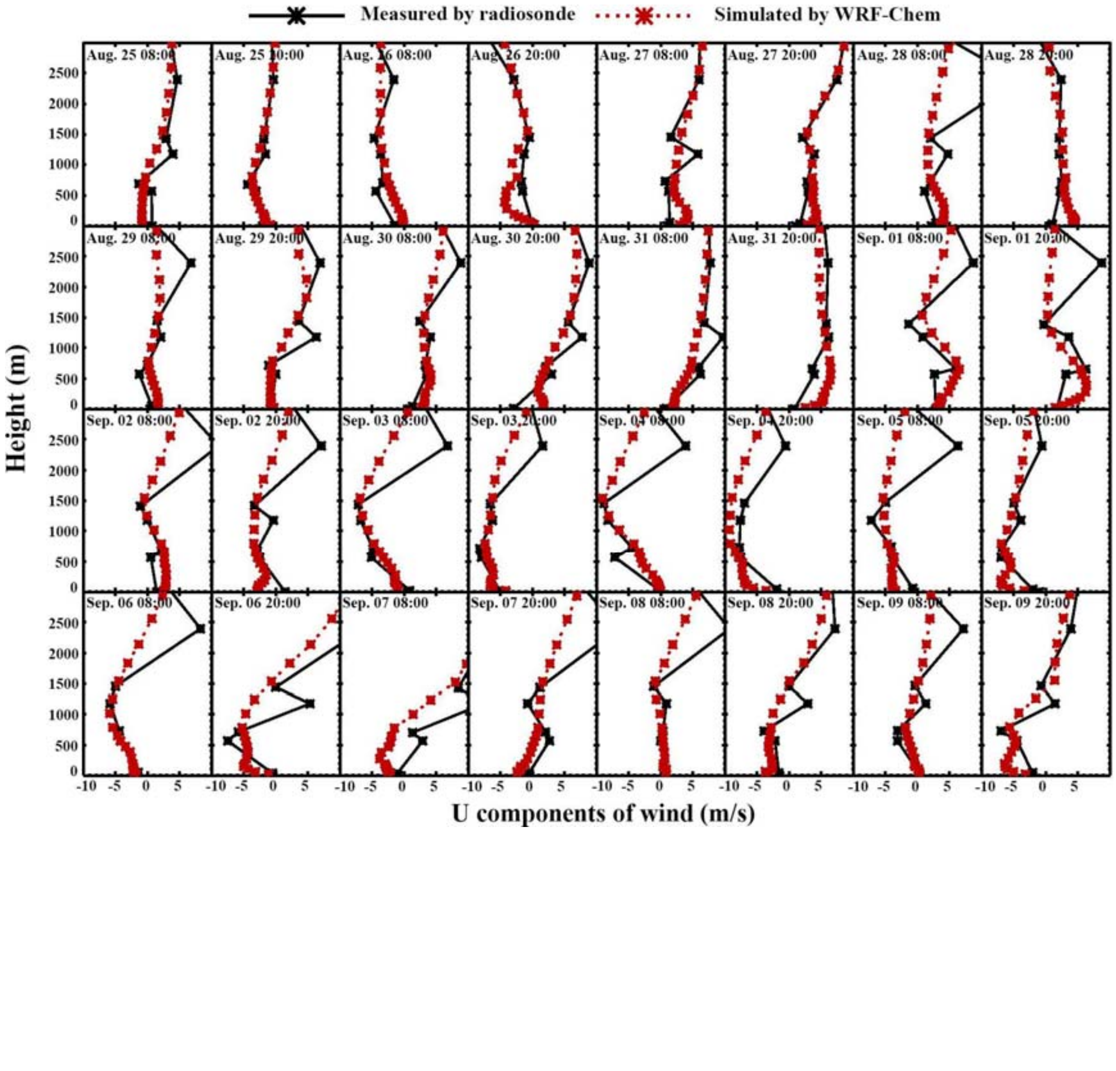
Measured by radiosonde
Simulated by WRF-Chem
Height (m)
U components of wind (m/s)

(e)

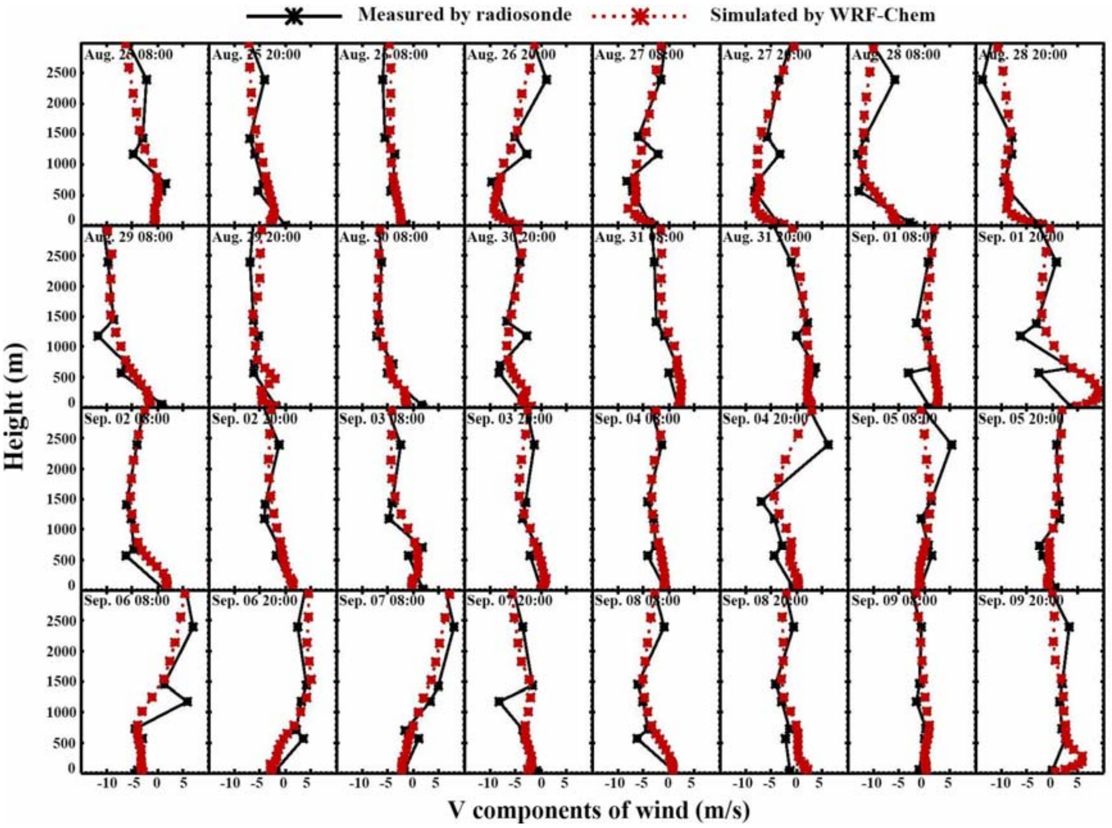

Figure S14. The vertical profiles of (a) temperature, (b) pressure, (c) water vapor mixing ratio, (d) zonal wind component (u), and (e) meridional wind component (v) simulated using the WRF-Chem model and observed by radiosondes.
